# Mapping the research landscape of UAV- and eVTOL-enabled emergency medical services: a bibliometric analysis, scoping review, and policy tool assessment

**DOI:** 10.3389/frhs.2026.1861375

**Published:** 2026-10-15

**Authors:** Zhiqiang Hao, Guoxuan Xu, Wei Zhuang, Liangru Xu, Tie Xu, Xingchen Zhu, Xianliang Yan

**Affiliations:** 1 Division of Planning and Academic Development, Xuzhou Medical University, Xuzhou, China; 2 Department of Emergency Medicine, The Affiliated Hospital of Xuzhou Medical University, Xuzhou, China; 3 The Laboratory of Emergency Medicine, The First Clinical Medical College of Xuzhou Medical University, Xuzhou, China; 4 Office of Hospital Director, The Affiliated Hospital of Xuzhou Medical University, Xuzhou, China; 5 Outpatient Department, The Affiliated Hospital of Xuzhou Medical University, Xuzhou, China

**Keywords:** bibliometric analysis, emergency medical services, eVTOL, policy analysis, unmanned aerial vehicle

## Abstract

**Background:**

Electric vertical takeoff and landing (eVTOL) aircraft and unmanned aerial vehicles (UAVs) are increasingly recognized as transformative technologies for emergency medical services (EMS). Despite a growing body of evidence on drone-delivered automated external defibrillators (AEDs), blood product logistics, and eVTOL air ambulance simulations, no study has comprehensively mapped this interdisciplinary research landscape while simultaneously analyzing the policy frameworks that govern its implementation.

**Methods:**

We conducted a triple-method study integrating bibliometric analysis, a scoping review following PRISMA-ScR guidelines, and quantitative policy text analysis. Bibliometric mapping covered 2015 to 2026. The scoping review followed the Joanna Briggs Institute methodology with the PAGER (Patterns, Advances, Gaps, Evidence, Recommendations) synthesis framework, including 168 studies. Policy analysis applied the Rothwell and Zegveld policy tool classification and the Policy Modeling Consistency (PMC) Index to 62 Chinese policy documents related to low-altitude economy and emergency medical rescue (2020–2025), yielding 526 codable policy provisions.

**Results:**

The bibliometric analysis identified 683 publications across five thematic clusters, with exponential growth from 2022 onward. The scoping review of 168 studies synthesized evidence showing drone-delivered AEDs arrived before ambulances in 67% of cases (median lead time 3 min 14 s), blood product drone delivery reduced transport time by 61%, and eVTOL air ambulance simulations halved advanced life support response times. All 964 modeled drone networks were cost-effective under model assumptions, improving OHCA survival by 21%–46%. Policy analysis of 62 Chinese documents revealed structural imbalance: supply-side and environmental tools constituted 75.3% of provisions while demand-side tools accounted for only 24.7%. The mean PMC Index was 7.46 (SD = 0.89), with timeliness (0.73) and policy measures (0.72) as the weakest dimensions.

**Conclusion:**

The evidence base for UAV/eVTOL-enabled emergency medical services is approaching the consistency needed to inform health systems planning, although no formal risk-of-bias appraisal was undertaken, and a critical disconnect persists between technological readiness and policy infrastructure. We propose a “dual-wheel drive, four-chain integration” framework encompassing scenario implementation and ecosystem cultivation across infrastructure, industrial innovation, institutional policy, and human capital dimensions. This framework offers a roadmap for translating fragmented pilot programs into sustainable, equity-enhancing emergency medical service systems.

## 1 Introduction

Emergency medical services face an enduring and universal challenge: the race against time. For conditions such as out-of-hospital cardiac arrest, severe trauma, and acute stroke, every minute of delay in definitive care translates into measurably worse patient outcomes. A Taipei registry study of 4,278 OHCA cases found that each minute of delayed advanced life support response reduced survival to discharge by 7% and favorable neurological outcome by 9% (1). Traditional ground-based emergency medical systems impose physical constraints that incremental improvements in ground transportation cannot overcome (2). Against this backdrop, electric vertical takeoff and landing (eVTOL) aircraft and unmanned aerial vehicles (UAVs) have emerged as potentially disruptive technologies for emergency medical service delivery (3, 4).

Unlike traditional helicopters, which require large helipads, impose high operational costs, and generate substantial noise, eVTOL platforms leverage electric propulsion systems that enable vertical operations from compact urban sites including rooftops and parking structures. Their cruise speeds of 150–300 km/h allow point-to-point transit within metropolitan areas in under 10 min, and a single eVTOL-equipped station can theoretically cover an area ten times larger than its ground-based counterpart (5, 6). The evidence base supporting aerial emergency medical applications has expanded substantially. In Sweden, Schierbeck and colleagues demonstrated through prospective observation that drone-delivered AEDs arrived before ambulances in 67% of suspected OHCA cases, with a clinically meaningful median lead time of 3 min and 14 s (7). Denmark replicated these findings with semi-autonomous drone operations in a real-world feasibility study (8), and Canada provided the most comprehensive cost-effectiveness modeling to date, showing that all 964 modeled drone network configurations produced acceptable incremental cost-effectiveness ratios with survival improvements of 21%–46% (9). Rwanda's national drone delivery program, operated by Zipline, has completed over two million medical deliveries and demonstrably reduced blood product waste by 67% and delivery times by 61% (10). At the eVTOL scale, simulation studies across Paris and Vancouver suggest that deploying even one or two air ambulances cut advanced life support response times from 21 min to 7–9 min (11).

Simultaneously, a powerful policy momentum has emerged, particularly in China where the concept of a “low-altitude economy” (di kong jing ji) has been elevated to national strategic priority. First mentioned in China's Government Work Report in March 2024, the low-altitude economy was further designated as an “emerging pillar industry” in the 2026 report, signaling an unprecedented convergence of industrial policy, technological maturation, and public safety imperatives. By mid-2025, twenty-nine Chinese provinces had incorporated low-altitude economic development into their planning frameworks, and Shenzhen had enacted the first comprehensive subnational legislation governing low-altitude economic activities. Despite this convergence of technological progress, clinical evidence, and policy attention, the research landscape remains fragmented in ways that impede systematic health service planning. Studies on drone AED delivery, blood product logistics, eVTOL air ambulance simulation, and helicopter emergency medical services (HEMS) outcomes largely exist in disconnected literature streams, with minimal cross-referencing or integrated synthesis. Existing bibliometric analyses have focused either on small drones broadly (12), drone healthcare delivery mapped through topic modeling (13), advanced air mobility in general (14), or HEMS in isolation (15), without mapping the specific intersection of aerial platforms and emergency medical care. A small number of scoping and systematic reviews have synthesized subsets of the evidence (16–18), but none has incorporated quantitative policy analysis to assess whether governance frameworks are keeping pace with technological capabilities.

Publications addressing eVTOL aircraft specifically in medical or health contexts remain very scarce, only nine across all databases searched (Section 3.1.1); thus, we deliberately define the field broadly, encompassing UAV-based medical delivery, eVTOL platforms, and, as comparator context, helicopter emergency medical services. This study addresses these gaps through a triple-method design that is, to our knowledge, the first of its kind in this domain. We integrate bibliometric landscape mapping to reveal the structural characteristics of the research field, a PRISMA-ScR-compliant scoping review to synthesize the substantive evidence across application domains, and quantitative policy text analysis using the Rothwell and Zegveld framework (19, 20) and PMC Index (21) to evaluate the policy environment. The thematic clusters identified bibliometrically supply the domains along which the scoping review is organized, the implementation barriers surfaced by the review structure the value-chain dimension of the policy coding, and the resulting policy-gap diagnosis is interpreted against the maturity of the clinical evidence. Drawing on this integrated evidence base, we propose a “dual-wheel drive, four-chain integration” framework that bridges the gap between scenario-level technological demonstrations and the systemic ecosystem cultivation necessary for sustainable, equitable emergency medical service transformation.

## 2 Methods

### 2.1 Study design

This study employs a triple-method design comprising bibliometric analysis, scoping review, and quantitative policy text analysis. The scoping review component adheres to the PRISMA Extension for Scoping Reviews (PRISMA-ScR) reporting checklist developed by Tricco et al. (22) and follows the Joanna Briggs Institute (JBI) methodology for scoping reviews, which builds upon the foundational framework established by Arksey and O'Malley (23) and refined by Levac et al. (24). The bibliometric component follows the emerging ScoRBA (Scoping Review Based on Bibliometric Analysis) methodology with the PAGER (Patterns, Advances, Gaps, Evidence, Recommendations) synthesis framework (25). The integrated approach follows the Bibliometric-Systematic Literature Review (B-SLR) guideline proposed by Marzi et al. (26). This review was not prospectively registered. PROSPERO does not accept scoping reviews, and no *a priori* protocol was deposited in a public repository; to mitigate the transparency limitations that follow from the absence of registration, the complete database-specific search strategies and the data charting framework are provided in the Supplementary Material.

Bibliometric analysis addresses the macro-structural question of how the field is organized and where it is moving; the scoping review addresses the substantive question of what the accumulated evidence shows within each identified domain; and the policy text analysis addresses the institutional question of whether governance frameworks are keeping pace with that evidence. The five bibliometric clusters (Section 3.1.4) supplied the organizing categories for the scoping review's four application domains, with helicopter EMS treated as comparator context within the patient-transport domain. The barriers and enablers charted in the review informed the five value-chain stages used as the Y dimension of the policy coding matrix (Section 2.4.2). Burst references identified through citation analysis (Section 3.1.5) were prespecified as anchor studies for the narrative synthesis. The Discussion then reads the three sets of findings as a single diagnostic chain.

### 2.2 Bibliometric analysis

#### 2.2.1 Data sources and search strategy

Publications were retrieved from two primary databases: the Web of Science Core Collection (WoSCC), including the Science Citation Index Expanded (SCIE), Social Sciences Citation Index (SSCI), and Emerging Sources Citation Index (ESCI); and Scopus, which provides broader coverage of engineering and technology journals. The Chinese National Knowledge Infrastructure (CNKI) and Wanfang Data were searched as supplementary sources for Chinese-language publications to capture the rapidly growing domestic literature on low-altitude economy applications. In addition, PubMed/MEDLINE was queried with an equivalent strategy. PubMed records were not merged into the bibliometric corpus, which derives exclusively from the four databases above after deduplication; they served instead as an independently indexed, MeSH-curated benchmark series for the temporal trend analysis reported in Section 3.1.1 and Figure 1, providing a conservative cross-check on annual publication counts. The search strategy combined controlled vocabulary and free-text terms organized into two concept blocks connected by Boolean AND operators: (a) aerial platform terms (eVTOL OR “electric vertical takeoff” OR “air taxi” OR UAV OR “unmanned aerial vehicle” OR drone OR “advanced air mobility” OR “urban air mobility”) AND (b) emergency medical terms (emergency medical OR EMS OR ambulance OR “cardiac arrest” OR AED OR defibrillator OR “medical rescue” OR “blood delivery” OR “organ transport” OR “prehospital” OR HEMS OR “air ambulance”). Searches were executed in title, abstract, and keyword fields (TS in the Web of Science; TITLE-ABS-KEY in Scopus; title/abstract fields in PubMed; subject fields in CNKI and Wanfang), with the final update completed in March 2026. Records were exported in RIS format and deduplicated in reference-management software through DOI matching and normalized title-author-year matching, followed by manual verification of residual near-duplicates; Chinese-language records were harmonized with the international set by translating title and keyword fields and standardizing institutional names prior to analysis. The complete database-specific strategies, field codes, limits, and deduplication procedure are reported in Supplementary Material S1. The search covered publications from January 2015 through March 2026, with no language restrictions.

**Figure 1:**
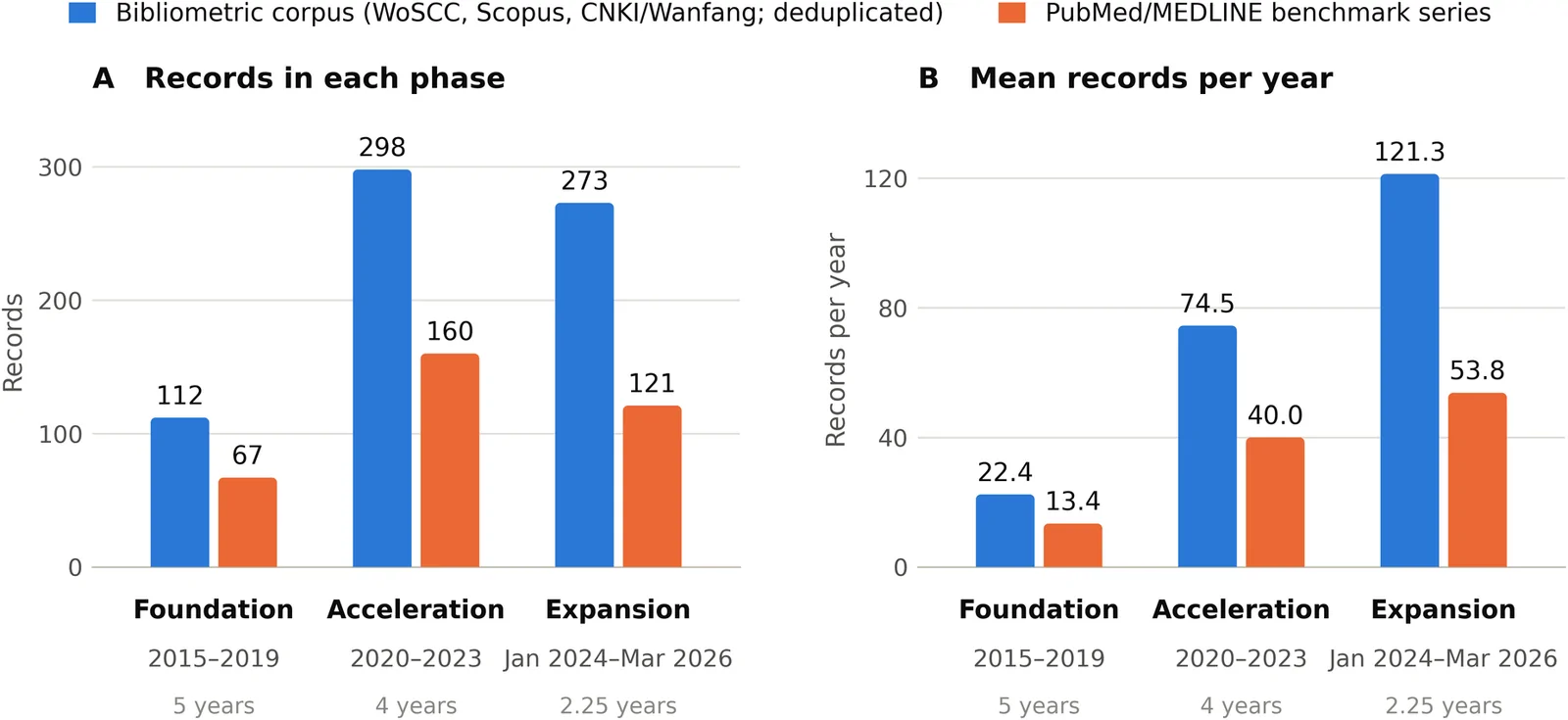
Growth of publications on UAV- and eVTOL-enabled emergency medical services across three developmental phases, January 2015 to March 2026. (A) Number of records in each phase. (B) Mean number of records per year, calculated by dividing each phase total by the length of the phase (5, 4, and 2.25 years). Blue bars represent the deduplicated bibliometric corpus (n = 683); orange bars represent the PubMed/MEDLINE benchmark series.

#### 2.2.2 Analytical tools and metrics

Three complementary bibliometric software tools were employed to ensure analytical robustness. VOSviewer (version 1.6.20, Centre for Science and Technology Studies, Leiden University) was used for constructing and visualizing co-authorship networks, keyword co-occurrence maps, and bibliographic coupling analyses, with fractional counting applied to normalize multi-authored publications. CiteSpace (version 6.3.R1, developed by Chaomei Chen, Drexel University) was used for citation burst detection, timeline visualization, and identification of intellectual turning points in the literature. The Bibliometrix R package (version 4.0, developed by Aria and Cuccurullo) (27) was used for comprehensive science mapping including Bradford's law analysis of core journals, Lotka's law assessment of author productivity, and thematic evolution mapping across defined time periods.

Key bibliometric indicators assessed include annual publication volume and growth rate, geographic distribution by country and institution, journal distribution patterns, co-citation networks to identify intellectual foundations, keyword dynamics to trace thematic evolution, and international collaboration patterns. Emerging research fronts were identified through CiteSpace's burst detection algorithm, which identifies references and keywords experiencing statistically significant increases in citation frequency within defined time windows.

For readers less familiar with these techniques, three constructs carry most of the interpretive weight in this study. A keyword co-occurrence cluster is a group of terms that repeatedly appear together in the same publications; clusters therefore approximate research communities rather than evidence categories. A citation burst identifies a reference whose citation rate rises sharply within a short window, signalling that the field has begun to organize around that study. Link strength between two clusters is a normalized measure of how often their keywords co-occur, so a low value indicates that two communities rarely address the same problems. All three are descriptive indicators of where scholarly attention is concentrated; they say nothing about the methodological quality of individual studies, which is why they are used here to structure the scoping review rather than to grade its evidence.

### 2.3 Scoping review

#### 2.3.1 Eligibility criteria

Studies were included if they met the following criteria based on the PCC (Population, Concept, Context) framework: Population: patients requiring emergency medical services or health systems providing such services; Concept: the use of UAVs, eVTOL aircraft, or other aerial platforms for any emergency medical application including but not limited to AED delivery, medical supply transport, patient transport, and search and rescue with medical components; Context: any geographic or health system setting, encompassing both high-income and low- and middle-income countries. All study designs were eligible, including randomized controlled trials, observational studies, simulation studies, modeling studies, cost-effectiveness analyses, qualitative studies, and mixed-methods research. Editorials, letters, conference abstracts without full text, and studies focused exclusively on non-medical drone applications (agricultural, military surveillance, recreational) were excluded.

#### 2.3.2 Study selection and data charting

Two reviewers independently screened titles and abstracts against the eligibility criteria, followed by full-text reviews of potentially eligible studies. Disagreements were resolved through discussion or, if necessary, adjudication by a third reviewer. A standardized data charting form was developed and pilot-tested on a random sample of 10 included studies before being applied to the full set. Charted variables included study characteristics (authors, year, country, design), aerial platform specifications (type, payload, range, speed), clinical application domain, outcome measures, key findings, and identified barriers and facilitators for implementation. The charting framework and study-level charting results for the included studies are provided in Supplementary Table S2.

#### 2.3.3 Synthesis framework

Evidence synthesis followed the PAGER framework: Patterns (recurring themes, dominant research paradigms, geographic concentrations), Advances (key breakthroughs and their implications for practice), Gaps (underexplored areas, methodological limitations, missing stakeholder perspectives), Evidence for Practice (findings with immediate relevance for health service planning), and Research Directions (priorities for future investigation). Studies were categorized into four primary application domains based on the aerial platform function: (1) automated external defibrillator delivery for out-of-hospital cardiac arrest; (2) medical supply and biological specimen transport; (3) patient transport and air ambulance services; and (4) health system integration and policy frameworks. Because a narrative synthesis of 168 studies cannot discuss every source individually, studies foregrounded in the text were selected against four prespecified salience criteria: real-world rather than exclusively simulated deployment; the largest sample, delivery volume, or number of modeled configurations within a domain; the availability of health-economic endpoints; and identification as burst references in the bibliometric component. The full evidence base underlying each domain is characterized in aggregate in Section 3.2.1, synthesized across domains in Section 3.2.6 and Table 1, and tabulated at study level in Supplementary Table S2.

**Table 1:** Cross-domain PAGER synthesis of the 168 included studies.

PAGER element	Cross-domain synthesis
Patterns	Research effort concentrates in two comparatively mature communities, drone AED delivery (42.9% of included studies) and medical supply logistics (22.6%), led by Sweden, Canada, Rwanda-based programs, and, increasingly, China; eVTOL (18.5%) and health-system/policy work (16.1%) form small, weakly connected communities; output accelerates sharply after 2022.
Advances	The decisive recent advances are economic and operational rather than narrowly technical: cost-effectiveness across all 964 modeled AED drone network configurations; real-world prospective and semi-autonomous deliveries in Sweden and Denmark; dispatcher-integration protocols raising bystander AED attachment from 27% to 50%; national-scale logistics operations exceeding two million deliveries.
Gaps	The deepest gaps are institutional rather than technological: reimbursement and procurement pathways, aviation-medical workforce categories, airspace governance for routine rather than demonstration operations, patient-level outcome data for eVTOL transport, and systematic evaluation of operator-held data; talent cultivation receives only 12.4% of policy value-chain coverage.
Evidence for practice	Network design methods, dispatcher protocols, and blood-logistics operating models are ready to inform structured pilots in systems with response-time gaps exceeding 8–10 min or blood-bank access beyond 30 min, provided deployment is coupled to prospective outcome evaluation.
Research directions	Priorities are outcome verification (survival, neurological outcome, maternal mortality) under routine operations; health-economic evaluation beyond modeled assumptions; equity analyses of coverage; governance and financing research bridging the eVTOL and policy clusters; and grey-literature synthesis conducted with operator cooperation.

#### 2.3.4 Critical appraisal

Consistent with the JBI guidance for scoping reviews and with the PRISMA-ScR checklist, in which critical appraisal of individual sources is an optional item (22), no formal methodological quality or risk-of-bias assessment was undertaken, because the objective was to map the extent, range, and nature of the evidence rather than to grade the certainty of effect estimates. First, the synthesis gives descriptive rather than weighted treatment to studies of differing rigor. Second, throughout the Results and Discussion we frame evidential claims in deliberately graded language: where terms such as “comparatively mature” appear, they denote the volume, replication, and design diversity of a body of literature.

### 2.4 Policy text analysis

#### 2.4.1 Policy document selection

A corpus of 62 policy documents was assembled through systematic retrieval from official Chinese government websites, including the State Council, National Development and Reform Commission, Ministry of Industry and Information Technology, Civil Aviation Administration of China, National Health Commission, and relevant provincial and municipal government portals, as well as the Peking University Law Database (PKULaw). Documents were included if they were issued between January 2020 and December 2025, promulgated by government agencies at or above the provincial level, and contained substantive provisions related to low-altitude economy (or specific eVTOL/UAV) development and emergency management or medical service delivery. Pure technical standards and internal administrative notices without policy directives were excluded. The final corpus comprised 26 national-level and 36 provincial-level documents, covering planning outlines (*n* = 8), guiding opinions (*n* = 11), implementation plans (*n* = 16), administrative measures and regulations (*n* = 7), and official notices (*n* = 20).

#### 2.4.2 Rothwell and Zegveld policy tool classification

Each policy document was coded using the Rothwell and Zegveld (1981, 1985) tripartite classification of policy instruments (19, 20). Supply-side tools encompass government actions that directly augment the capacity for innovation and service delivery, including four subcategories: financial investment (fiscal appropriations, special funds), infrastructure construction (vertiport networks, communication and navigation systems), talent cultivation (discipline development, training bases), and information technology support (digital platforms, intelligent dispatch systems). Environmental tools create enabling conditions through four subcategories: laws and regulations (legislation, technical standards), airspace management (airspace classification, approval reform), tax incentives (exemptions, industry subsidies), and safety regulation (airworthiness certification, operational oversight). Demand-side tools stimulate market formation and uptake through four subcategories: government procurement (service contracts, public welfare projects), demonstration applications (pilot cities, scenario expansion), international cooperation (standard harmonization, technical exchange), and public education (publicity, social mobilization). This 3-category, 12-subcategory system provides comprehensive coverage of the policy instrument spectrum in low-altitude emergency medical rescue.

Two researchers independently coded all 62 documents at the clause level using the directed content analysis approach described by Hsieh and Shannon (28), with each policy provision assigned to both the X dimension (policy tool type) and the Y dimension (emergency rescue value chain stage: technology R&D, infrastructure, talent cultivation, operational assurance, and application promotion). Inter-rater reliability was assessed using Cohen's kappa coefficient, yielding *κ* = 0.83, indicating excellent agreement (*κ* ≥ 0.75). Disagreements were resolved by a third researcher, producing a final cross-tabulation matrix of 526 valid coding units.

#### 2.4.3 PMC index model

The Policy Modeling Consistency (PMC) Index, developed by Estrada (2011) (21), was applied to evaluate the comprehensiveness and internal consistency of individual policy documents. A multi-input-output table was constructed with nine primary variables and 35 secondary variables tailored to the low-altitude economy emergency medical rescue domain: policy nature (X1: supervisory, predictive, guiding), policy scope (X2: economic, safety, science and technology), policy timeliness (X3: long-term, medium-term, short-term), policy objectives (X4: safety assurance, industrial development, public service, technological innovation), policy content (X5: technology R&D, infrastructure, talent cultivation, operational management, application promotion), policy targets (X6: government, enterprises, social organizations, the public), policy evaluation (X7: sufficient basis, clear objectives, clear planning, scientific scheme), policy measures (X8: fiscal support, institutional development, talent assurance, technology support, regulatory optimization), and policy instruments (X9: supply-side, environmental, demand-side, compound). Each secondary variable was coded as binary (1 = addressed, 0 = not addressed). PMC scores were calculated as the sum of nine primary variable scores (each being the arithmetic mean of its secondary variables), yielding values on a 0–9 scale where 8–9 indicates “Excellent,” 7–7.99 “Good,” 5–6.99 “Acceptable,” and below 5 “Poor.” Three-dimensional PMC surface diagrams were generated for representative policies to visualize dimensional strengths and weaknesses.

## 3 Results

### 3.1 Bibliometric overview

#### 3.1.1 Publication trends and growth patterns

The search strategy yielded 1,185 records across all databases (WoSCC: 423; Scopus: 582; CNKI/Wanfang: 145; reference scanning: 35). After deduplication, 683 unique publications remained for bibliometric analysis (Figure 2). Throughout this section, corpus counts refer to these 683 deduplicated records; PubMed figures denote the benchmark series described in Section 2.2.1 and are reported in parentheses for cross-validation only. Annual publication volume exhibited a characteristic exponential growth curve, with a sharp inflection point observed from 2022 onward. The period from 2015 to 2019 produced approximately 112 unique corpus records (67 in the PubMed benchmark series), representing the foundational phase during which early feasibility studies and simulation models predominated. The acceleration phase from 2020 to 2023 saw annual output roughly triple, coinciding with the emergence of real-world prospective studies from Sweden and operational programs from Rwanda, yielding approximately 298 corpus records (160 in the benchmark series). Most notably, the 2024–2026 period witnessed unprecedented growth with approximately 273 corpus records (121 in the benchmark series) in just over two years, reflecting both the maturation of the drone AED evidence base and global policy momentum around advanced air mobility. China's domestic publications experienced particularly dramatic growth during this final period, driven by the national strategic prioritization of the low-altitude economy. Only 9 publications across all databases addressed eVTOL aircraft specifically in the context of medical or health applications, confirming the nascent but rapidly emerging nature of this subfield and motivating the broad platform scope adopted in this study (Section 1).

**Figure 2:**
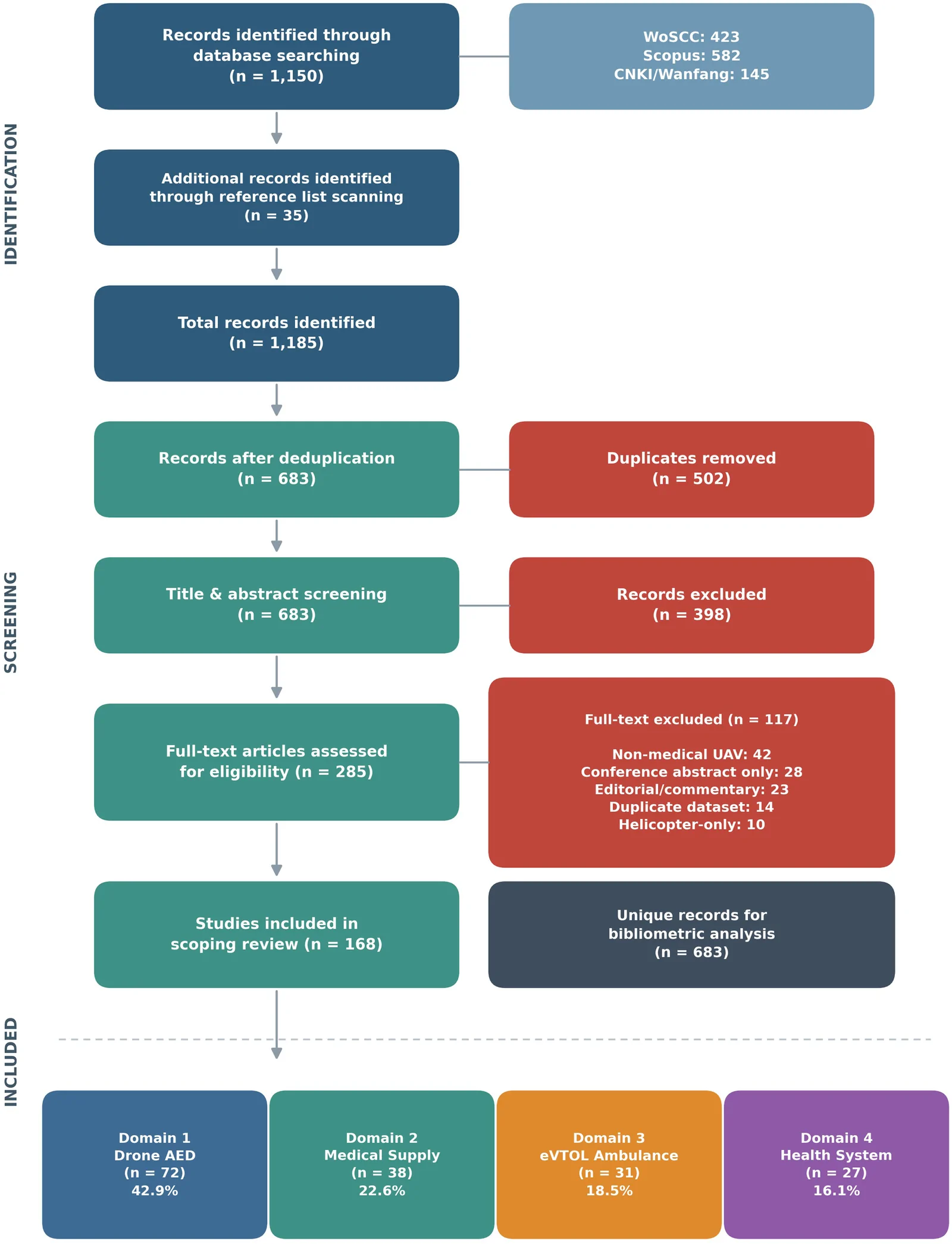
PRISMA-ScR flow diagram for study selection. Database searching identified 1,150 records (Web of Science Core Collection, *n* = 423; Scopus, *n* = 582; CNKI/Wanfang, *n* = 145), and reference list scanning contributed an additional 35 records, for a total of 1,185 records identified. After removal of 502 duplicates, 683 unique records underwent title and abstract screening, of which 285 proceeded to full-text assessment. Following exclusion of 117 articles for specified reasons (non-medical UAV application, *n* = 42; conference abstract only, *n* = 28; editorial/commentary without original data, *n* = 23; duplicate dataset reporting, *n* = 14; traditional helicopter-only without novel technology comparison, *n* = 10), 168 studies were included in the scoping review and categorized into four application domains: drone AED delivery (*n* = 72, 42.9%), medical supply transport (*n* = 38, 22.6%), eVTOL air ambulance (*n* = 31, 18.5%), and health system integration and policy (*n* = 27, 16.1%). All 683 deduplicated records were retained for bibliometric analysis. The flow diagram follows the PRISMA Extension for Scoping Reviews (PRISMA-ScR) reporting guidelines.

#### 3.1.2 Geographic and institutional distribution

Research output was concentrated in a relatively small number of countries. The United States, Sweden, China, Canada, and Germany collectively accounted for approximately 72% of all publications. Sweden's disproportionate influence reflects the Karolinska Institutet group's pioneering prospective studies of drone-delivered AEDs (7, 29), while the University of Toronto has established itself as the leading center for drone network optimization and cost-effectiveness modeling (9). China's research contribution has grown rapidly from 2024 but remains predominantly in domestic journals, underscoring the importance of the CNKI/Wanfang supplementary search in this study.

#### 3.1.3 Journal distribution and core sources

Bradford's law analysis identified a core zone of 12 journals that published the majority of highly cited work. Resuscitation and its companion journal Resuscitation Plus emerged as the most prolific venues, collectively publishing 38 articles, consistent with the field's strong orientation toward cardiac arrest applications. The Lancet Digital Health published the highest-impact individual study (Schierbeck et al., 2023), while the New England Journal of Medicine contributed the seminal case report of the first drone-delivered AED survival (29). The Lancet Global Health served as the primary outlet for evidence from low- and middle-income country implementations, most notably the Rwanda blood delivery evaluation (10). JAMA and its specialty journals contributed foundational HEMS evidence (30). Engineering-focused journals including Applied Sciences and Drones contributed feasibility analyses, while the Scandinavian Journal of Trauma, Resuscitation and Emergency Medicine published the largest HEMS meta-analysis to date (31).

#### 3.1.4 Keyword co-occurrence and thematic clusters

VOSviewer keyword co-occurrence analysis, applying a minimum threshold of 5 occurrences, revealed five distinct thematic clusters (Figure 3). Cluster 1 (Drone AED Delivery), the largest and most densely interconnected, centered on keywords including “out-of-hospital cardiac arrest,” “automated external defibrillator,” “drone delivery,” “response time,” and “bystander defibrillation.” Cluster 2 (Medical Supply Logistics) incorporated “blood delivery,” “supply chain,” “rural health,” and “cost-effectiveness,” anchored by the Rwanda Zipline evidence. Cluster 3 (eVTOL and Advanced Air Mobility) featured “eVTOL,” “urban air mobility,” “air ambulance,” “airworthiness,” and “vertiport,” representing an emergent frontier with relatively few publications and strong recent growth. Cluster 4 (Helicopter EMS) comprised the traditional HEMS literature with keywords such as “helicopter,” “trauma,” “survival,” and “prehospital care.” Cluster 5 (Health System and Policy Integration) was the smallest but fastest-growing cluster, featuring “health policy,” “regulation,” “airspace management,” and “implementation science.” The linkage density between Clusters 3 and 5 was notably weak (link strength = 0.12, compared to 0.67 between Clusters 1 and 2), confirming the disconnect between eVTOL technological research and health governance scholarship that motivated this study.

**Figure 3:**
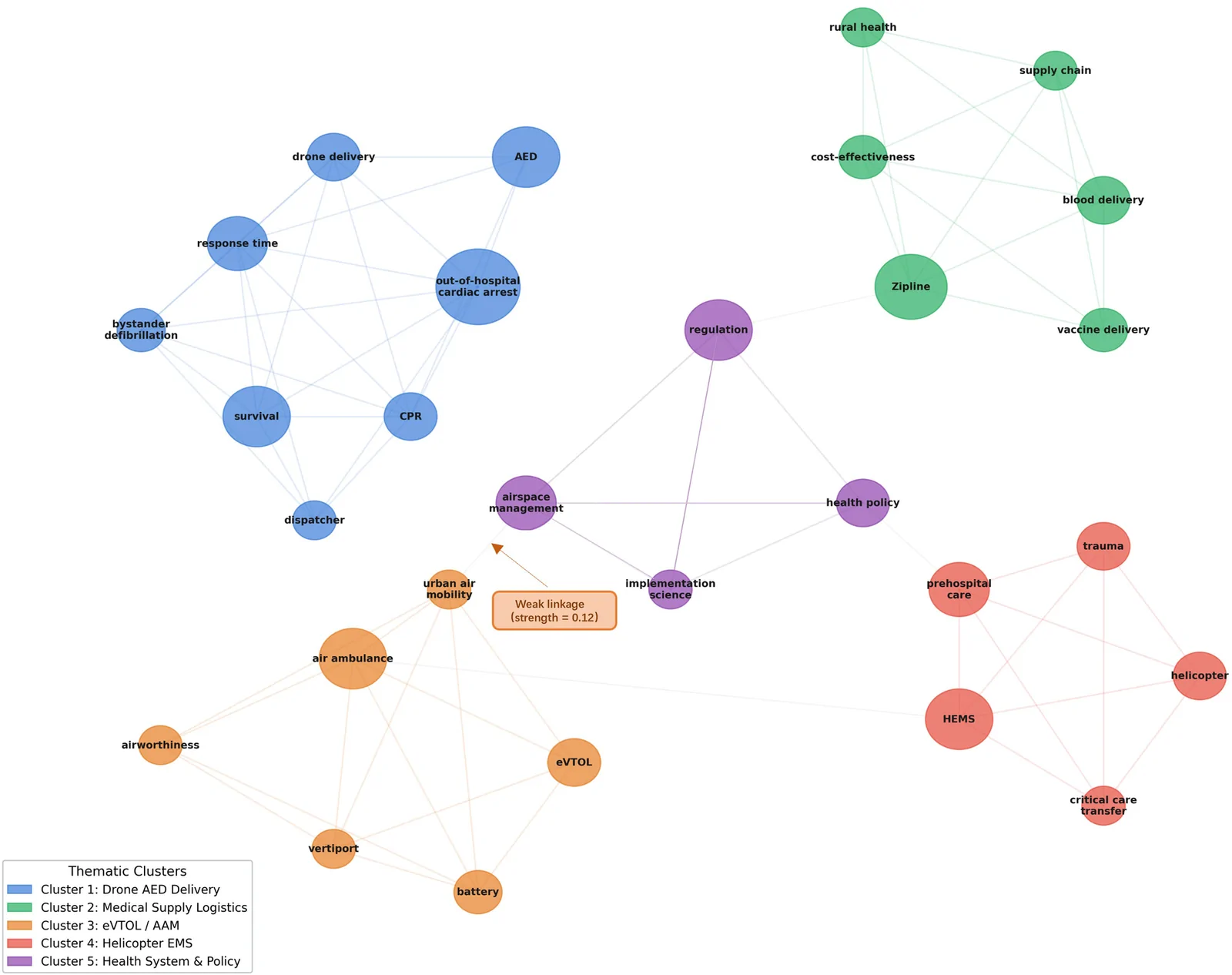
Simplified keyword co-occurrence network derived from the VOSviewer analysis, showing representative keywords of the five thematic clusters. Five thematic clusters were identified through automated clustering with association strength normalization. Cluster 1 (blue, Drone AED Delivery) represents the most mature and densely interconnected research domain, centered on out-of-hospital cardiac arrest, automated external defibrillator, and response time. Cluster 2 (green, Medical Supply Logistics) encompasses blood delivery, supply chain, and cost-effectiveness, anchored by the Rwanda Zipline evidence. Cluster 3 (orange, eVTOL and Advanced Air Mobility) features eVTOL, urban air mobility, and airworthiness as an emergent frontier with the fewest publications but strongest recent growth. Cluster 4 (red, Helicopter EMS) comprises the traditional HEMS literature. Cluster 5 (purple, Health System and Policy Integration) is the smallest but fastest-growing cluster. Node size is proportional to keyword occurrence frequency, and link thickness reflects co-occurrence strength. The notably weak linkage between Cluster 3 and Cluster 5 (strength = 0.12, compared to 0.67 between Clusters 1 and 2) confirms the disconnect between eVTOL technological research and health governance scholarship that motivated this study's integrated design.

#### 3.1.5 Citation burst and emerging frontiers

CiteSpace burst detection analysis identified 14 references with statistically significant citation bursts (Figure 4). The strongest and most recent bursts were associated with Schierbeck et al. (2023) on real-world drone AED delivery (burst strength = 8.73, 2023–2026), Maaz et al. (2025) on drone network cost-effectiveness (burst strength = 6.91, 2025–2026), and Heidet et al. (2023) on eVTOL air ambulance simulation (burst strength = 5.44, 2024–2026). Keywords experiencing the most recent bursts included “eVTOL” (2024–2026), “cost-effectiveness analysis” (2025–2026), “low-altitude economy” (2025–2026), and “health equity” (2025–2026), indicating a research front that is shifting from proof-of-concept studies toward health economic evaluation and systems integration. The temporal co-citation analysis revealed a clear evolutionary trajectory from the HEMS foundations (pre-2015), and the network-optimization lineage inaugurated by Boutilier et al. (2017) (32), through the drone AED feasibility phase (2016–2021), into the current evidence consolidation and eVTOL expansion phase (2022–present).

**Figure 4:**
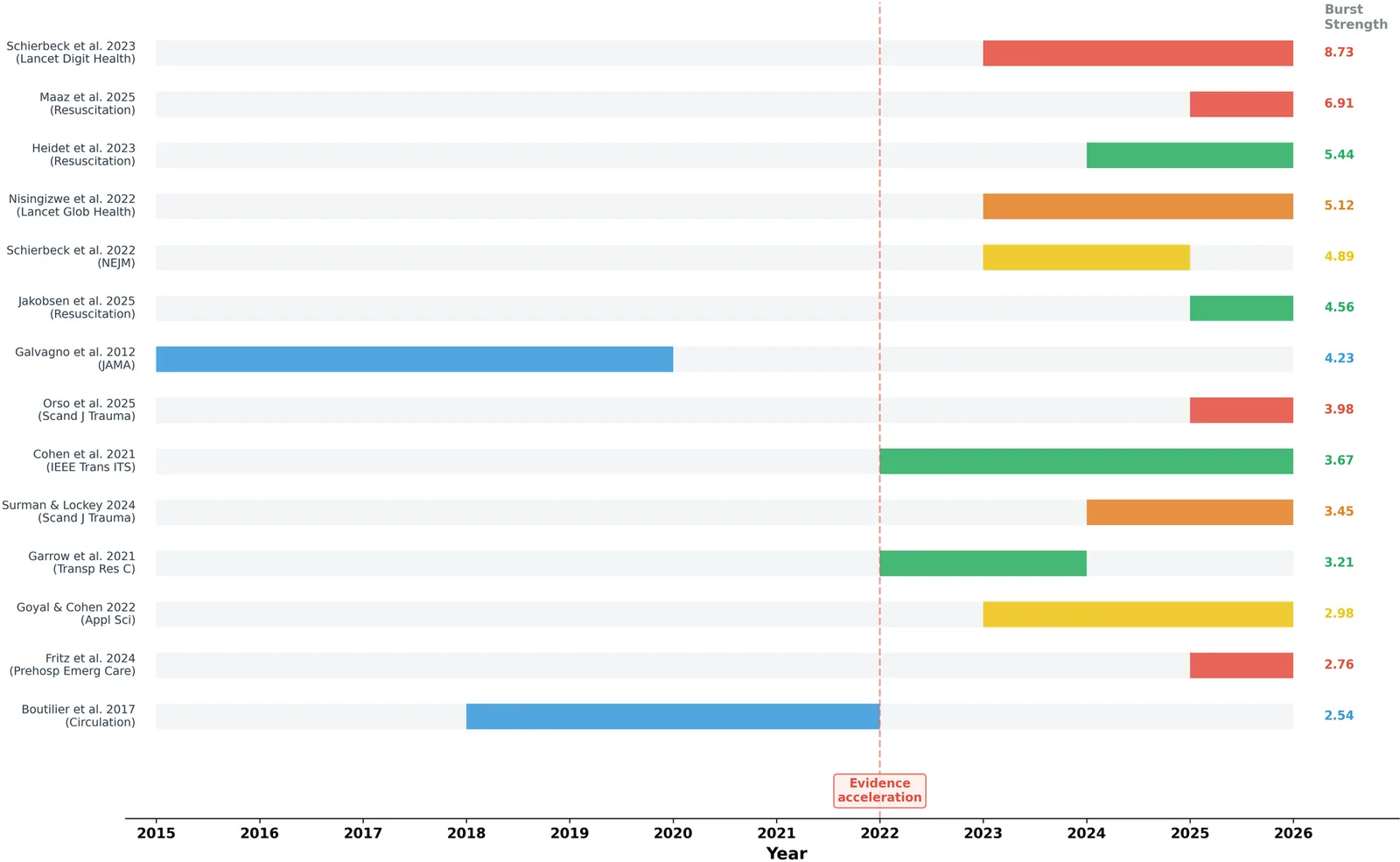
CiteSpace citation burst timeline for top 14 references*.* Horizontal bars indicate the burst period for each reference; the two blue bars mark foundational references, and the other colors serve only to distinguish individual references. Burst strength values are displayed to the right of each bar. Schierbeck et al. (2023, The Lancet Digital Health) exhibited the strongest burst (8.73), followed by Maaz et al. (2025, Resuscitation; burst strength 6.91) and Heidet et al. (2023, Resuscitation; 5.44). The vertical dashed line at 2022 marks the evidence acceleration inflection point, after which publication volume and citation activity increased sharply. The temporal distribution of bursts reveals a clear evolutionary trajectory from foundational HEMS evidence (Galvagno et al., 2012) and early drone-network optimization (Boutilier et al., 2017), through the drone AED feasibility phase, into the current evidence consolidation and eVTOL expansion phase dominated by cost-effectiveness analyses and health system integration studies.

### 3.2 Scoping review findings

#### 3.2.1 Study selection

The PRISMA-ScR flow diagram (Figure 2; Table 2) documents the screening process. Database searching yielded 1,150 records (WoSCC, 423; Scopus, 582; CNKI/Wanfang, 145), and reference list scanning contributed a further 35 records, for a total of 1,185 records identified. After removal of 502 duplicates, 683 unique records underwent title and abstract screening. Title and abstract screening excluded 398 records, and full-text assessment of 285 articles led to the exclusion of 117 articles for the following reasons: non-medical UAV application (*n* = 42), conference abstract only without full-text access (*n* = 28), editorial or commentary without original data (*n* = 23), duplicate reporting of the same dataset (*n* = 14), and exclusive focus on traditional helicopter without comparative or novel technology component (*n* = 10). The final scoping review included 168 studies published between 2015 and March 2026 (Table 3).

**Table 2:** PRISMA-ScR flow diagram summary.

Screening Stage	*n*
Records identified through database searching	1,150
Web of Science Core Collection	423
Scopus	582
CNKI/Wanfang	145
Additional records identified through reference list scanning	35
**Total records identified**	**1,185**
Duplicates removed	502
Records after deduplication	683
Records excluded at title/abstract screening	398
Full-text articles assessed for eligibility	285
Full-text articles excluded (with reasons)	117
Non-medical UAV application	42
Conference abstract only	28
Editorial/commentary without original data	23
Duplicate dataset reporting	14
Traditional helicopter only without novel comparison	10
**Studies included in scoping review**	**168**
**Unique records included in bibliometric analysis**	**683**

**Table 3:** Summary of key evidence by application domain.

Application Domain	Key Study	Design	Key Finding	n Studies
Drone AED Delivery	Schierbeck et al. 2023 (7)	Prospective observational	67% arrived before ambulance; median lead 3:14	72 (42.9%)
Drone AED Delivery	Maaz et al. 2025 (9)	Markov microsimulation	All 964 networks cost-effective under model assumptions; 21%–46% survival gain	—
Blood/Supply Delivery	Nisingizwe et al. 2022 (10)	Retrospective cohort	61% faster delivery; 67% less waste	38 (22.6%)
eVTOL Air Ambulance	Heidet et al. 2023 (11)	Simulation cohort	ALS time: 21→7–9 min (Paris)	31 (18.5%)
HEMS (context)	Orso et al. 2025 (31)	Systematic review/MA	Mortality RR 1.13 (0.96–1.34); very low quality	—
Health System/Policy	—	Mixed	Regulatory uncertainty as top barrier	27 (16.1%)

The included studies were heterogeneous in design, setting, and analytic tradition. The evidence base spans prospective observational deployments, retrospective operational cohorts, case reports, Markov and discrete-event microsimulations, geographic information system-based network optimizations, cost-effectiveness and cost-utility analyses, qualitative and mixed-methods studies of bystander and dispatcher experience, and regulatory or implementation analyses. Geographically, it is distributed across high-income settings in Europe and North America, upper-middle-income settings led by China, and low- and middle-income operational programs concentrated in sub-Saharan Africa, with publication activity heavily concentrated after 2022 in a pattern mirroring the bibliometric inflection. The distribution across the four application domains, 72 studies (42.9%) on drone AED delivery, 38 (22.6%) on medical supply and specimen transport, 31 (18.5%) on eVTOL patient transport and air ambulance services, and 27 (16.1%) on health system integration and policy, itself constitutes a finding: a maturity gradient runs from the first domain to the fourth, and the gradient structures the synthesis below. Study-level characteristics are tabulated in Supplementary Table S2. The subsections that follow synthesize each domain in turn, foregrounding studies that meet the prespecified salience criteria described in Section 2.3.3.

#### 3.2.2 Domain 1: drone-delivered AEDs for out-of-hospital cardiac arrest

This domain constituted the most mature evidence base, comprising 72 of the 168 included studies (42.9%). The landmark Swedish prospective observational study by Schierbeck et al. (2023, The Lancet Digital Health) deployed drones to 55 suspected OHCA cases in the Gothenburg region, finding that drones arrived before ambulances in 67% of cases with a median time advantage of 3 min and 14 s (7). A subsequent analysis from the same group demonstrated that a structured educational bundle for dispatchers could substantially improve bystander engagement with drone-delivered AEDs, increasing the AED attachment rate from 27% to 50% (33). Denmark's feasibility study by Jakobsen et al. (2025, Resuscitation) deployed semi-autonomous drones in Aalborg, achieving a 100% delivery success rate across 16 eligible cases with no adverse events, although the median drone response time of 4 min 47 s was slightly slower than the corresponding ambulance response of 3 min 25 s in that urban context (8), a finding that highlights the critical dependence of drone effectiveness on base station placement relative to existing EMS infrastructure density.

The economic evidence from this domain is particularly compelling. Building on the network-optimization lineage established by Boutilier and colleagues, whose combined optimization-queuing model of 53,702 Toronto-area cardiac arrests first demonstrated that mathematically designed drone networks could systematically shorten AED arrival (32), Maaz et al. (2025, Resuscitation) constructed a Markov microsimulation model using data from 22,017 OHCAs over a decade in Ontario, Canada, evaluating 964 distinct drone network configurations (9). Every configuration evaluated proved cost-effective within the model's assumptions, with the smallest non-dominated network (20 drones) generating an incremental cost-effectiveness ratio of $20,912 per quality-adjusted life year gained. Drone networks reduced response times by 32%–71% compared to standard care and improved hospital discharge survival by 21%–46%. In China, peer-reviewed deployment evidence remains at an early stage: a GIS-based analysis has optimized AED drone deployment networks within the walking golden window for Xuzhou (34), while prospective comparative pilots in several Chinese cities have been registered but not yet reported (Section 4.5).

#### 3.2.3 Domain 2: medical supply and biological specimen transport

This domain comprised 38 of the 168 included studies (22.6%). Rwanda's Zipline program provides the most extensive operational evidence for drone-based medical logistics. The analysis by Nisingizwe et al. (2022, The Lancet Global Health) covering 12,733 deliveries documented a 61% reduction in blood product delivery time and a 67% decrease in blood unit expiration rates (10). As of 2026, Zipline has expanded to six countries with over two million deliveries. Analyses conducted with the Rwandan Ministry of Health have linked the program to a 51% reduction in in-hospital maternal mortality from postpartum hemorrhage; because these analyses have so far been reported only outside the peer-reviewed literature, we treat this figure as grey-literature evidence awaiting independent confirmation (Section 4.5). In China, SF Express launched Wuhan's first drone blood distribution route in December 2024, delivering 800 mL of blood in 19 min, though peer-reviewed outcomes data from these programs remain limited.

#### 3.2.4 Domain 3: eVTOL patient transport and air ambulance services

Evidence in this domain, comprising 31 of the 168 included studies (18.5%), remains predominantly simulation-based, reflecting the early stage of eVTOL certification for passenger-carrying operations. The most rigorous study is Heidet et al. (2023, Resuscitation), which modeled 6,217 real OHCA cases across Greater Paris and Metro Vancouver (11). Deploying one to two VTOL ambulances reduced advanced life support arrival times from 21 min to 7–9 min in Paris and from 12 to 7–9 min in Vancouver, improvements that proved statistically significant across all evaluated metrics. Goyal and Cohen (2022, Applied Sciences) provided a multi-criteria feasibility analysis identifying battery technology as the primary constraint: current charge times would need a four-fold reduction before eVTOLs could compete operationally with ground ambulances for short-distance transports under 40 kilometers (35). Industry partnerships are advancing rapidly, with ADAC Luftrettung/Volocopter planning 250 multicopter stations across Germany and several Chinese manufacturers developing medical-specific eVTOL platforms, at estimated operating costs one-third to one-half those of conventional helicopters.

An important contextual finding emerged from the traditional HEMS literature. The largest meta-analysis to date by Orso et al. (2025, Scandinavian Journal of Trauma, Resuscitation and Emergency Medicine), pooling 77 studies involving 2.6 million patients, found that HEMS did not demonstrably improve survival compared to ground EMS, with a mortality risk ratio of 1.13 (95% CI: 0.96–1.34) based on very low quality evidence, and the total incremental net benefit was estimated at approximately EUR 980,000 per quality-adjusted life year (31). Subgroup evidence complicates rather than resolves this picture: a focused meta-analysis restricted to patients with an Injury Severity Score of at least nine found a survival benefit for helicopter scene response (36), suggesting that aerial platforms may generate value in well-selected populations even where all-comer analyses are null. This uncertain cost-effectiveness of traditional HEMS creates a powerful argument for novel eVTOL platforms that could potentially deliver comparable or superior clinical capabilities at substantially lower per-mission costs.

#### 3.2.5 Domain 4: health system integration and policy frameworks

This domain represented the smallest body of evidence, comprising 27 of the 168 included studies (16.1%). The available literature consists primarily of perspective pieces, regulatory analyses, and implementation frameworks rather than empirical evaluations. Existing reviews have consistently identified regulatory uncertainty, airspace integration, public acceptance, and workforce readiness as the principal barriers to routine aerial EMS operations (16, 17). De Silvestri et al. (2023) found that only one study in their scoping review of healthcare drone challenges substantively addressed regulatory frameworks, and that study was limited to Polish and European Union law (16). Zhu, Xu, and Yan (2026) provided the first systematic exploration of eVTOL-enabled emergency medical rescue system construction within China's “Fifteenth Five-Year Plan” framework, analyzing specific scenarios for high-rise buildings, mountainous terrain, subterranean spaces, and open waterways (3). The Chinese literature, while growing rapidly, has predominantly appeared in domestic emergency medicine and aviation management journals, where it documents early institutional experimentation, including proposals for accelerating an aviation medical emergency rescue system (37), county-level air rescue practice in the mountainous areas of southwestern Zhejiang (38), and evaluations of air-ground integrated response for critically ill patients in remote areas (39), with limited engagement with the international health services research community.

#### 3.2.6 Cross-domain PAGER synthesis

Applying the PAGER framework across the four domains yields the integrated synthesis summarized in Table 1. Stated compactly, the pattern is one of asymmetric maturation. The field's empirical center of gravity lies in drone AED delivery and medical logistics; its most consequential recent advances are economic and operational rather than narrowly technical; its deepest gaps are institutional, concerning governance, reimbursement, and workforce, rather than technological; its practice-ready evidence concerns network design, dispatcher integration, and delivery logistics; and its research agenda is visibly shifting from feasibility demonstration toward outcome verification, health-economic evaluation, and equity analysis. This synthesis supplies the empirical warrant for the framework developed in Section 4.2.

### 3.3 Policy analysis results

#### 3.3.1 Rothwell and Zegveld classification

Content analysis of the 62 policy documents at the clause level yielded 526 valid coding units (Table 4; Figure 5). The distribution across the three policy tool categories revealed a pronounced structural imbalance. Environmental tools were the most prevalent, with 202 coding units (38.4%), predominantly concentrated in laws and regulations (71 units, 35.1% within category) and airspace management reform (53 units, 26.2%), reflecting the “institution-first” logic characteristic of China's approach to nascent industries. Supply-side tools ranked second with 194 coding units (36.9%), led by financial investment (62 units, 32.0%) and infrastructure construction (58 units, 29.9%), indicating substantial government commitment to hardware development including vertiport networks and communication systems. Demand-side tools were markedly underrepresented with only 130 coding units (24.7%), of which government procurement (42 units, 32.3%) and demonstration applications (38 units, 29.2%) were the dominant subcategories, while international cooperation (24 units, 18.5%) and public education (26 units, 20.0%) received minimal attention.

**Table 4:** Distribution of policy tool types.

Policy Tool Type	Subcategory	Frequency	Within-type %	Overall %
Supply-side (*n* = 194, 36.9%)	Financial investment	62	32.0	11.8
	Infrastructure construction	58	29.9	11.0
	Talent cultivation	36	18.6	6.8
	IT support	38	19.6	7.2
Environmental (*n* = 202, 38.4%)	Laws and regulations	71	35.1	13.5
	Airspace management	53	26.2	10.1
	Tax incentives	32	15.8	6.1
	Safety regulation	46	22.8	8.7
Demand-side (*n* = 130, 24.7%)	Government procurement	42	32.3	8.0
	Demonstration applications	38	29.2	7.2
	International cooperation	24	18.5	4.6
	Public education	26	20.0	4.9

**Figure 5:**
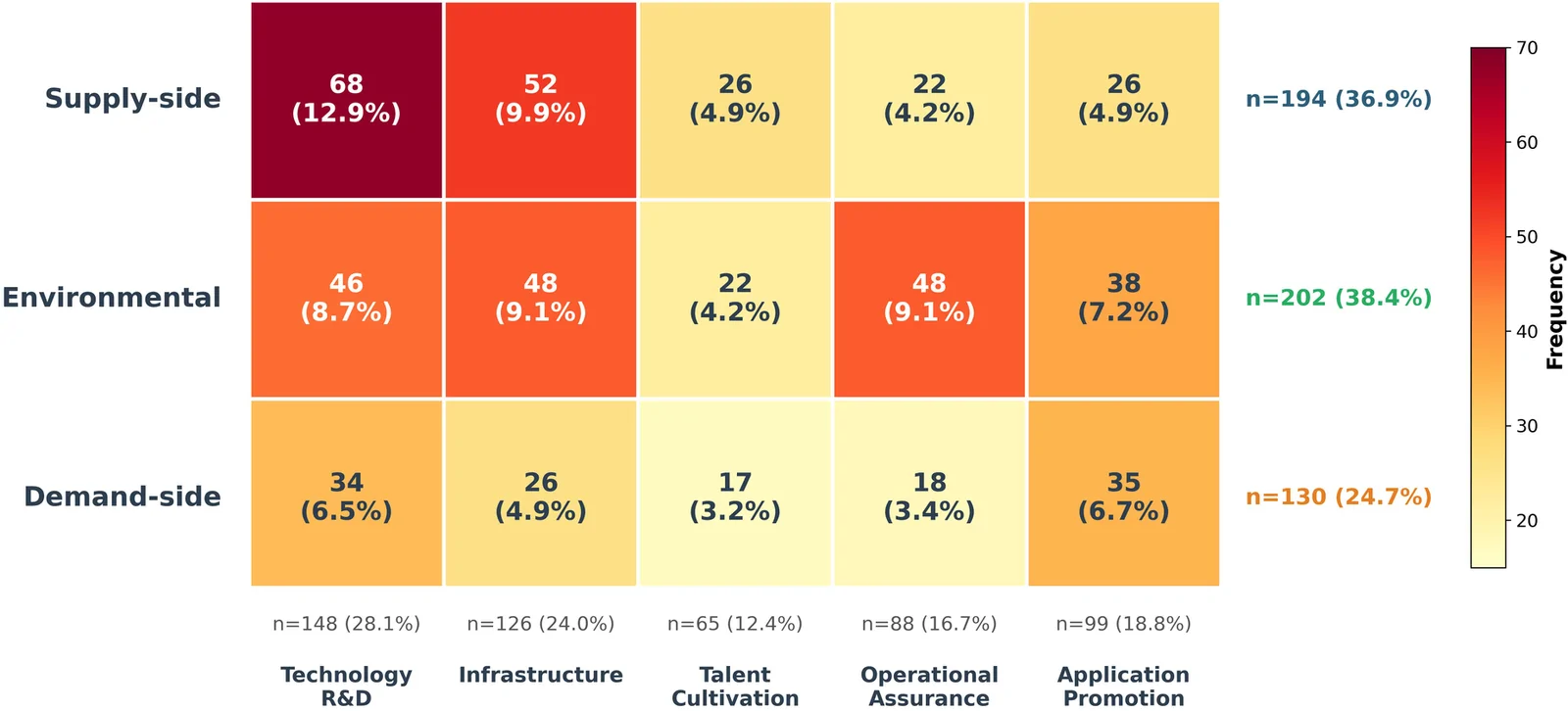
Rothwell and Zegveld policy tool distribution across the emergency rescue value chain. The heatmap displays the frequency of coded policy provisions at the intersection of three policy tool types (rows: supply-side, environmental, demand-side) and five value chain stages (columns: technology R&D, infrastructure, talent cultivation, operational assurance, application promotion). Cell values indicate absolute frequency with overall percentage in parentheses; color intensity corresponds to frequency magnitude. Row and column marginal totals with percentages are displayed along the right and bottom edges. The distribution reveals a “heavy on supply and environment, light on demand” structural pattern, with supply-side (36.9%) and environmental (38.4%) tools collectively accounting for 75.3% of all provisions, while demand-side tools constitute only 24.7%. Along the value chain dimension, technology R&D (28.1%) and infrastructure (24.0%) dominate, whereas talent cultivation receives markedly less policy attention (12.4%). Inter-rater reliability for the coding process achieved Cohen's *κ* = 0.83.

This “heavy on supply and environment, light on demand” distribution pattern closely parallels findings from other Chinese strategic emerging industry policy analyses, including new energy vehicles and artificial intelligence, and from health policy domains such as integrated care, where demand-side tools have been consistently identified as the structural weak link. From the emergency rescue value chain perspective, technology R&D received the highest policy density (148 units, 28.1%), followed by infrastructure (126 units, 24.0%), application promotion (99 units, 18.8%), and operational assurance (88 units, 16.7%). Talent cultivation received the lowest policy attention with only 65 coding units (12.4%), highlighting a critical gap in the training of hybrid aviation-medical professionals that is particularly problematic for eVTOL emergency medical operations.

#### 3.3.2 PMC index evaluation

PMC scores across the 62 documents ranged from 5.28 to 8.72, with a mean of 7.46 (SD = 0.89), classified as “Good” on the Estrada scale (Table 5). National-level documents issued by the State Council and multi-ministry joint publications scored highest (mean = 8.14, SD = 0.52), reflecting their broader scope and greater comprehensiveness across evaluation dimensions. Provincial implementation opinions typically scored in the “Good” range (mean = 7.21, SD = 0.76), while documents focused narrowly on either aviation or emergency management (but not both) showed the greatest variability. The PMC surface diagrams (Figure 6) revealed consistent patterns across the corpus: most documents performed well on policy nature (X1, mean = 0.89), policy objectives (X4, mean = 0.86), and policy scope (X2, mean = 0.84), but showed notable depressions in the timeliness dimension (X3, mean = 0.73) and policy measures dimension (X8, mean = 0.72). The policy instruments dimension (X9, mean = 0.78) reflected the demand-side deficit identified in the Rothwell and Zegveld analysis. This pattern suggests that low-altitude economy policies have been designed primarily from an industrial development perspective, with insufficient attention to the specific timeliness requirements, concrete implementation measures, and health system integration mechanisms that would be necessary for emergency medical applications.

**Table 5:** PMC index scores by dimension (*N* = 62 policy documents).

PMC Dimension	Variable	Mean Score	Interpretation
X1 Policy nature	Supervisory, predictive, guiding	0.89	Strong
X2 Policy scope	Economic, safety, S&T	0.84	Strong
X3 Policy timeliness	Long/medium/short term	0.73	Needs improvement
X4 Policy objectives	Safety, industry, public service, innovation	0.86	Strong
X5 Policy content	R&D, infrastructure, talent, operations, promotion	0.81	Good
X6 Policy targets	Government, enterprises, social orgs, public	0.79	Good
X7 Policy evaluation	Basis, objectives, planning, scheme	0.82	Good
X8 Policy measures	Fiscal, institutional, talent, tech, regulatory	0.72	Needs improvement
X9 Policy instruments	Supply, environmental, demand, compound	0.78	Good
**Overall PMC Index**	—	**7.46 (SD 0.89)**	Good (range 5.28–8.72)

**Figure 6:**
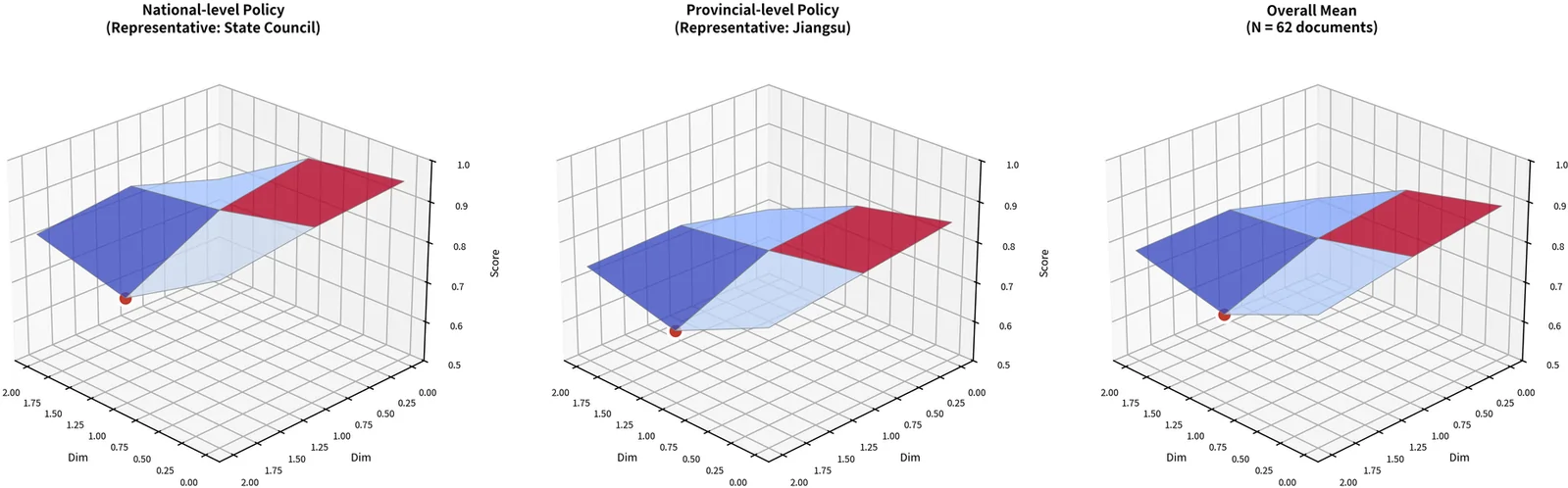
Three-dimensional PMC surface diagrams for representative national and provincial policies. Three panels display PMC surfaces for a national-level policy (left, State Council document), a provincial-level policy (center, Jiangsu Province implementation opinion), and the overall mean across all 62 documents (right). Each surface is constructed from nine primary evaluation dimensions (X1 policy nature through X9 policy instruments) arranged in a 3 × 3 grid, with the z-axis representing dimension scores (range 0–1). A red dot marks the lowest-scoring dimension in each panel. Representing the weakest scoring areas. Across all three panels, consistent depressions are observed in the timeliness dimension (X3, overall mean = 0.73) and policy measures dimension (X8, mean = 0.72), indicating that policies tend to establish broad strategic directions without specifying concrete implementation timelines or operational mechanisms. The overall PMC mean of 7.46 (SD = 0.89) falls within the “Good” classification on the Estrada scale (7.00–7.99), with national-level documents scoring higher (mean = 8.14) than provincial-level documents (mean = 7.21).

## 4 Discussion

### 4.1 Principal findings and interpretation

This study presents the first integrated bibliometric, scoping review, and policy analysis of UAV- and eVTOL-enabled emergency medical services, revealing a field undergoing rapid maturation in its evidence base but facing a critical policy-implementation gap. Because the three components were designed as a sequence rather than as parallel exercises, their findings can be read as a single diagnostic chain: the bibliometric map shows a field whose technological and governance conversations barely touch; the scoping review shows clinical evidence outrunning institutional readiness within that field; and the policy analysis locates the institutional deficit with precision, in demand-side activation, implementation timelines, and human capital. Before developing the three principal findings, we state in plain terms where the literature stands as of the March 2026 search.

Five statements summarize that position. First, drone delivery of AEDs is the only application for which real-world prospective deployment data, replicated feasibility studies on three continents, and comprehensive cost-effectiveness modeling now coexist; this evidence supports structured and evaluated pilots in systems with response-time gaps of 8–10 min or more, but survival benefit remains model-based rather than demonstrated in controlled studies. Second, drone medical logistics has the largest operational footprint of any application and the thinnest peer-reviewed evaluation base; a small number of national programs, above all Rwanda's, supply most of the outcome evidence, and the literature lags field practice by years. Third, eVTOL patient transport rests on simulation and feasibility analysis; none of the included studies reports an actual patient transported by an eVTOL platform, and the helicopter literature against which eVTOL will be judged shows that time savings do not automatically translate into survival. Fourth, governance, financing, and workforce questions form the smallest and least empirical body of literature, and the policy corpus we analysed is weighted toward supply-side and environmental instruments; the binding constraint on the field is now institutional rather than technological. Fifth, relative to the 2024 baseline of Campbell et al. (13), the literature has moved from asking whether drones can deliver to asking whether health systems can govern, purchase, and staff what they deliver (Section 4.3), while the published record continues to under-represent operator-led activity (Section 4.5). Three principal findings underpin these statements, with direct implications for health service planning, research prioritization, and governance innovation.

First, the bibliometric analysis of 683 publications demonstrates that the field has transitioned from a nascent exploration phase to one approaching sufficient evidentiary density for health systems decision-making. The exponential publication growth from 2022 onward, driven by landmark prospective studies and comprehensive cost-effectiveness analyses, indicates that the fundamental question is no longer whether aerial platforms can contribute to emergency medical care but rather how health systems should integrate them. The identification of five distinct thematic clusters with weak inter-cluster linkage, particularly between the eVTOL technology cluster and the health system policy cluster (link strength = 0.12), reveals a structural fragmentation that impedes translational progress. This structural fragmentation, with the two communities publishing in different journals and operating within distinct intellectual frameworks, demands deliberate interdisciplinary bridge-building of the kind this study attempts.

Second, the scoping review of 168 studies reveals an asymmetric evidence landscape. The drone AED delivery domain has achieved a level of evidence consistency (including real-world prospective data from Sweden and Denmark, replicated feasibility demonstrations across three continents, and cost-effectiveness modeling showing all 964 tested configurations viable under model assumptions) that can plausibly inform health system adoption planning, with the caveat that no formal risk-of-bias appraisal was performed (Section 2.3.4). The medical supply logistics domain similarly benefits from extensive operational data, particularly the Rwanda Zipline program with over two million deliveries and grey-literature indications of reduced maternal mortality. By contrast, the eVTOL air ambulance domain remains at the simulation stage, though the quality and geographic diversity of available simulations are improving. The traditional HEMS literature provides a sobering context: despite decades of operational experience and billions of dollars in investment, the largest meta-analysis to date (77 studies, 2.6 million patients) could not conclusively demonstrate survival benefits over ground EMS (mortality RR 1.13, 95% CI: 0.96–1.34), a finding that simultaneously validates the search for more cost-effective aerial platforms and cautions against assuming that faster response times automatically translate into better outcomes.

Third, the policy analysis of 526 coded provisions across 62 documents identifies a structural mismatch between the policy tool mix and the current needs of the field. The dominance of supply-side (36.9%) and environmental (38.4%) tools, while understandable for an emerging technology requiring research investment and regulatory frameworks, leaves a critical gap in demand-side activation (24.7%). Governments have been effective at funding research, building infrastructure, and establishing safety standards, but have devoted insufficient attention to creating the procurement mechanisms, insurance pathways, and market signals that would incentivize sustained operational deployment. The PMC analysis reinforces this finding: while the overall mean score of 7.46 indicates generally sound policy design, the consistently low scores in timeliness (0.73) and policy measures (0.72) indicate that policies tend to set broad directions without specifying the concrete implementation timelines and operational mechanisms needed for real-world deployment.

### 4.2 The “dual-wheel drive, four-chain integration” framework

Synthesizing insights from the bibliometric mapping, evidence review, and policy analysis, we propose a “dual-wheel drive, four-chain integration” framework (Figure 7) for advancing eVTOL-enabled emergency medical services from fragmented pilot programs toward sustainable health system integration. This framework distinguishes two complementary but distinct strategic imperatives, scenario implementation and ecosystem cultivation, that must advance in parallel rather than sequentially.

**Figure 7:**
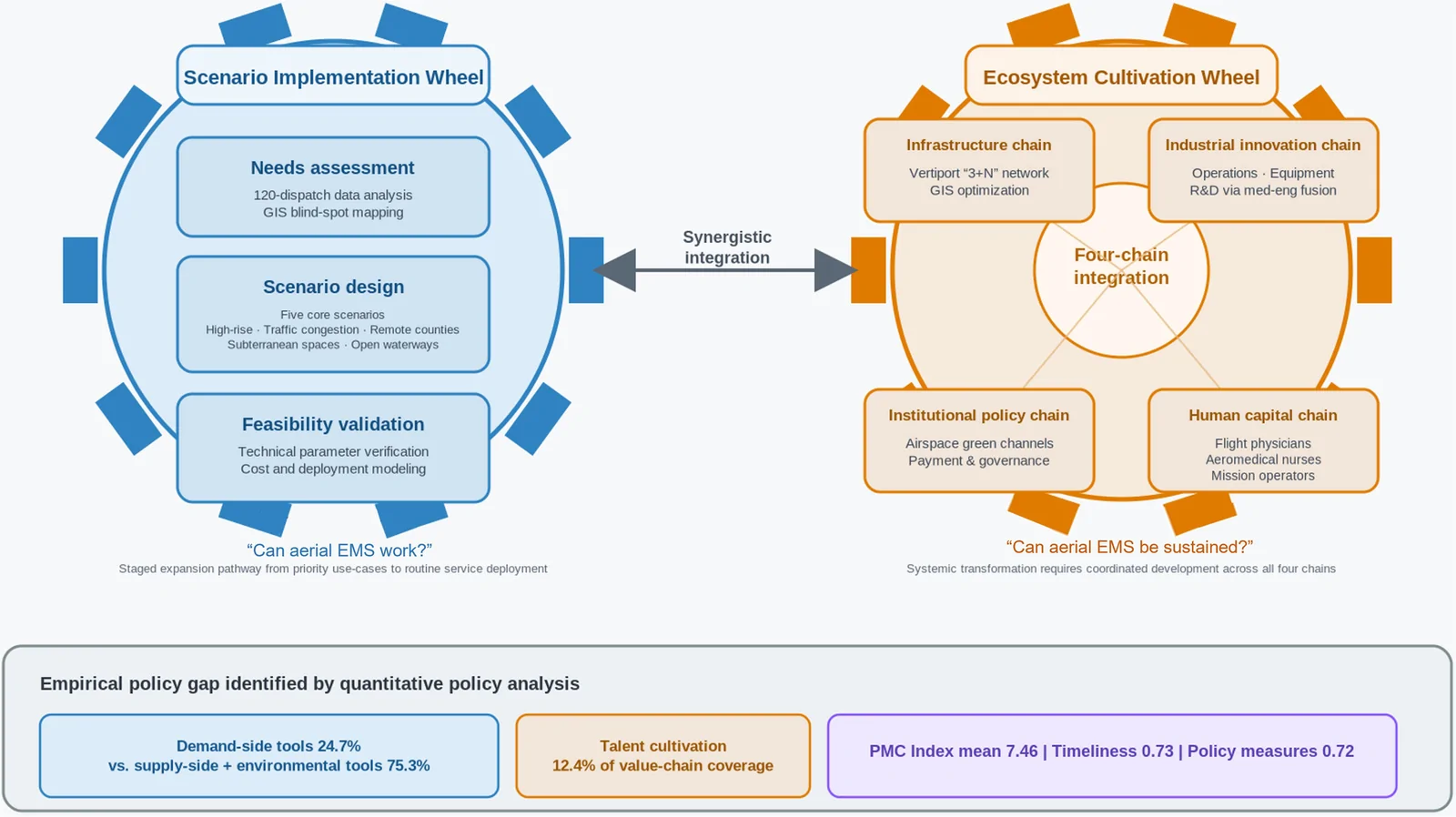
Conceptual diagram of the “dual-wheel drive, four-chain integration” framework. The framework comprises two synergistically linked strategic imperatives. The Scenario Implementation Wheel (left) addresses “whether aerial EMS can work” through needs assessment (120 dispatch data analysis, GIS blind-spot mapping), scenario design (five core scenarios covering high-rise buildings, traffic congestion, remote counties, subterranean spaces, and open waterways, with a staged expansion pathway), and feasibility validation (technical parameter verification and cost modeling). The Ecosystem Cultivation Wheel (right) addresses “whether aerial EMS can be sustained” through four interdependent chains: the infrastructure chain (vertiport “3 + N” network with GIS optimization), the industrial innovation chain (operations, equipment manufacturing, and technology R&D through “med-eng fusion”), the institutional policy chain (airspace green channels, diversified payment mechanisms, and cross-sector governance), and the human capital chain (flight physicians, aeromedical nurses, and eVTOL medical mission operators). The central bidirectional arrow indicates the synergistic integration between the two wheels. The bottom panel highlights the policy gap identified through quantitative analysis: demand-side tools account for only 24.7% of policy provisions while supply-side and environmental tools constitute 75.3%, and talent cultivation represents only 12.4% of emergency rescue value chain coverage. Key PMC Index metrics (mean = 7.46, timeliness = 0.73, policy measures = 0.72) are annotated to ground the framework in the empirical policy findings.

The scenario implementation wheel addresses the question of whether aerial emergency medical services can work in specific operational contexts. It encompasses needs assessment to identify the geographic, temporal, and clinical conditions under which aerial platforms offer meaningful advantages over ground-based services, and scenario design to develop operationally viable models for priority applications. The evidence synthesized in this review suggests that several scenario types have reached or are approaching implementation readiness: drone AED delivery for OHCA in areas with response times exceeding 8–10 min; blood product and medical supply delivery to facilities more than 30 min from blood banks; and eVTOL patient transport for time-sensitive conditions across distances of 40–150 kilometers where ground transport times are clinically meaningful.

The ecosystem cultivation wheel addresses the qualitatively different question of whether aerial emergency medical services can be sustained, scaled, and equitably distributed. Our analysis identifies four interdependent chains whose coordinated development is necessary for systemic transformation. The two-wheel logic draws on scene-driven innovation theory, which holds that application scenarios are not merely test sites but generative institutions that organize technology, capital, and governance around concrete demands (40), and on scholarship on the modernization of emergency management systems and capacity, which frames institutional development as co-equal with technical capability rather than derivative of it (41). The infrastructure chain encompasses vertiport networks and communication systems designed on a dual-use principle. The industrial innovation chain spans operational services, specialized medical device manufacturing, and enabling technology R&D. The institutional policy chain covers airspace green channels, diversified payment mechanisms, and cross-sector governance. The human capital chain addresses the training of hybrid aviation-medical professionals, a workforce category that does not yet exist in any country's occupational classification system.

The framework's central argument is that the failure mode for eVTOL emergency medical services is unlikely to be technological but rather ecosystemic. A successful demonstration flight does not constitute a sustainable service; it merely proves that one link in a complex chain can function under controlled conditions. Without local operational capacity, government procurement contracts, insurance reimbursement, and trained personnel, even technically mature aerial medical platforms will remain confined to showcase events. The policy analysis findings directly support this interpretation: the heavy emphasis on supply-side and environmental tools (75.3% combined) reflects a governance approach oriented toward enabling individual demonstrations rather than cultivating the systemic conditions for routine operations, while the deficit in demand-side tools (24.7%) represents the specific policy gap that must be addressed for the ecosystem cultivation wheel to turn.

### 4.3 Comparison with previous reviews and change since 2024

Our findings both extend and challenge the conclusions of prior reviews in this space. The most instructive comparison is with Campbell and colleagues, who in mid-2024 applied topic modeling to 290 drone healthcare delivery publications and identified two largely disconnected research themes, drones delivering time-critical medical items in response to emergencies and drones delivering non-time-critical items for supply chain resilience, with regulatory and implementation questions underdeveloped in both (13). Two years on, our five-cluster map registers both continuity and movement relative to that baseline. The bifurcation they observed persists as our Clusters 1 and 2, still the two largest communities. But three developments postdate their search window. The economic question they flagged as open has been substantially addressed for drone AED networks by comprehensive cost-effectiveness modeling (9); real-world operational evidence has thickened, from semi-autonomous deliveries in Denmark (8) to dispatcher-integration data from Sweden (33); and two clusters barely visible in 2024, eVTOL/advanced air mobility and health system and policy integration, have emerged as identifiable communities, albeit connected by the weakest linkage in the entire map (0.12). The gap identified in 2024 has therefore migrated rather than closed: from whether drones can deliver, to whether health systems can govern, purchase, and staff what drones deliver. The scale of movement is itself notable: the roughly 273 corpus records added between 2024 and the March 2026 search approach the 290 articles that constituted the entire 2010–2024 base analysed by Campbell et al., although the two counts are not directly comparable, since their scope covered healthcare delivery beyond emergency care and ours is confined to emergency medical applications.

The bibliometric component also advances beyond Pulsiri and Vatananan-Thesenvitz (2021), whose combined bibliometric-systematic review covered only 40 publications from a single database during a period that predates the current evidence acceleration (12), and beyond single-method mappings confined to traditional HEMS (15) or to advanced air mobility without a medical focus (14). The scoping review of 168 studies complements the recent systematic review by Zhang et al. (2026) of UAVs in prehospital emergency care covering 53 studies (17) and the scoping review of drone-delivered AEDs commissioned by the International Liaison Committee on Resuscitation (18), while substantially expanding the scope to include eVTOL platforms, health economic evidence, and policy dimensions that were outside the purview of those reviews.

Our cost-effectiveness synthesis arrives at a more optimistic assessment than some prior analyses because we incorporate the 2025 Maaz et al. data showing cost-effectiveness across all 964 drone network configurations under the model's assumptions (9). Previous reviews conducted before this study was available could not make definitive statements about the economic viability of drone EMS networks. At the same time, the inclusion of the Orso et al. (2025) HEMS meta-analysis introduces important nuance: if traditional helicopter EMS cannot conclusively demonstrate survival benefits in the largest pooled analysis ever conducted (31), then the threshold for demonstrating the value of eVTOL platforms should focus not merely on response time improvements but on whether those time savings translate into clinically meaningful outcome differences through paradigms such as “bringing hospital capacity to the scene” rather than merely “transporting patients faster” (3).

### 4.4 Implications for health service planning and policy

For health service planners, the evidence supports structured, piloted incorporation of drone AED delivery into OHCA response protocols for regions with existing response time gaps exceeding 8–10 min, provided that investments in bystander education and dispatcher protocols accompany the technological deployment and that deployment is coupled to prospective outcome evaluation. The cost-effectiveness data are of a scale and consistency that merit consideration in health technology assessment processes in most jurisdictions, with the caveat that the underlying primary studies were not formally appraised for risk of bias. For eVTOL air ambulance services, the simulation evidence justifies the initiation of real-world pilot programs with rigorous prospective evaluation protocols, particularly for inter-facility critical care transfers across distances of 50–150 kilometers where ground transport times are clinically meaningful.

For policymakers, the Rothwell and Zegveld analysis points to a specific actionable recommendation: governments should rebalance their policy tool mix by increasing the proportion of demand-side instruments from the current 24.7% toward a more balanced distribution. Concrete measures include government procurement contracts with guaranteed minimum activity levels, insurance mandates for medically justified aerial transport, demonstration subsidies of sufficient duration (12–24 months rather than single flights) to generate outcomes data, and patient access equity standards to prevent aerial EMS from exacerbating geographic disparities in emergency care. Framing these instruments through the lens of public value creation clarifies their purpose (42): demand-side tools do not so much subsidize an industry as constitute a public service market in which reliability, equity of access, and accountability are contractually specified. The PMC Index findings further suggest that policies should be strengthened in their timeliness (mean 0.73) and policy measures (mean 0.72) dimensions, moving from broad directional statements toward specific implementation timelines and operational protocols, particularly regarding the integration of aviation safety requirements with medical emergency response procedures.

### 4.5 Published evidence and field practice

Aerial medical logistics is an operator-led domain in which evidence generation lags practice by years rather than months. Zipline began routine national blood delivery in Rwanda in 2016, yet the first comprehensive peer-reviewed evaluation appeared only in 2022 (10), six years and hundreds of thousands of flights later; the reported 51% reduction in in-hospital maternal mortality from postpartum hemorrhage, derived from analyses with the Rwandan Ministry of Health, remains outside the peer-reviewed literature altogether. The same pattern recurs elsewhere. ADAC Luftrettung's multicopter program in Germany, SF Express's blood distribution routes in Wuhan, urban drone delivery networks operated by Chinese logistics platforms, and several municipal AED drone pilots are generating operational data that has not yet reached academic venues, and prospective comparative pilots registered in Chinese cities remain unreported.

First, the landscape mapped in this study should be read as a lower bound on field activity: publication counts under-represent operational maturity, particularly for logistics applications and for China. Second, the asymmetry is itself diagnostic. Domains anchored in public research institutions, such as drone AED delivery in Scandinavia and Canada, publish early and often, while operator-led domains publish late and selectively, which risks biasing syntheses toward academically convenient applications and away from the largest real-world deployments. Third, the demand-side policy instruments whose scarcity we document offer a direct remedy: government procurement contracts and demonstration subsidies can be conditioned on registry participation, standardized outcome reporting, and data-sharing requirements, converting operational activity into evaluable evidence. We did not systematically search grey literature, industry reports, or government evaluations, and we identify such a synthesis, ideally conducted with operator cooperation, as a priority for future work in this field.

### 4.6 Limitations

Several limitations warrant acknowledgment. First, while the bibliometric analysis drew on two major databases plus Chinese supplementary sources, yielding 683 unique publications, it may not capture all relevant grey literature, industry reports, and government evaluations not indexed in academic databases; this omission is likely to be consequential in an operator-led field, and the published record mapped here should accordingly be read as a conservative lower bound on activity. Second, the scoping review methodology, by design, does not assess the quality of individual studies through formal risk-of-bias evaluation, meaning that the evidence synthesis gives equal weight to studies of varying methodological rigor; we have moderated evidential language throughout rather than attaching formal certainty grades. Third, the review was not prospectively registered, and although the salience criteria governing which studies are foregrounded in the narrative were prespecified, their application necessarily involves judgment. Fourth, the Rothwell and Zegveld classification, while well-established and achieving excellent inter-rater reliability (kappa = 0.83), involves subjective judgment in assigning individual policy provisions to categories. Fifth, the PMC Index, though widely applied in Chinese policy research, has not been specifically validated for aviation or emergency medical contexts. Sixth, the policy corpus was limited to Chinese national and provincial documents; comparative analysis with European, North American, or other Asian policy environments would strengthen the generalizability of the policy findings. Seventh, the study's title foregrounds eVTOL although eVTOL-specific medical publications remain few (*n* = 9); we retain the term to designate the emergent frontier toward which the broader UAV and HEMS evidence converges, but the synthesis necessarily rests principally on that broader base. Finally, the rapid pace of development means that the literature landscape may shift meaningfully between search completion (March 2026) and publication.

## 5 Conclusion

This study provides the first integrated bibliometric analysis (683 publications), scoping review (168 studies), and quantitative policy assessment (62 documents, 526 coding units) of UAV and eVTOL applications in emergency medical services. The evidence for drone-delivered AEDs in out-of-hospital cardiac arrest and for drone-based medical supply logistics has reached a level that supports active integration into health service delivery models: prospective real-world data confirm time advantages over conventional ambulance response, and comprehensive cost-effectiveness analyses demonstrate that all tested drone network configurations produce acceptable incremental cost-effectiveness ratios with survival improvements of 21%–46%. The eVTOL air ambulance domain, though still at the simulation stage, shows sufficient promise to justify carefully designed real-world pilot programs with embedded evaluation, particularly for inter-facility transfers across distances of 50–150 kilometers.

The quantitative policy analysis reveals that the current Chinese policy environment, despite producing 526 identifiable policy provisions with a mean PMC score of 7.46 (“Good”), is structurally weighted toward supply-side (36.9%) and environmental (38.4%) instruments at the expense of demand-side tools (24.7%) essential for translating technological availability into sustainable, equitable service delivery. This imbalance is compounded by relative weakness in the timeliness and concrete policy measures dimensions, and by the near-absence of policy attention to aviation-medical human capital development (talent cultivation constituting only 12.4% of value chain coverage).

The “dual-wheel drive, four-chain integration” framework proposed in this study offers an analytical and practical architecture for navigating this transition. Its central insight is that sustainable aerial emergency medical services require not merely successful technology demonstrations but the deliberate cultivation of an ecosystem encompassing infrastructure, industrial capacity, institutional governance, and human capital. As nations worldwide accelerate their investment in advanced air mobility, the health services research community has both an opportunity and a responsibility to ensure that the integration of these technologies into emergency care systems is guided by evidence, equity, and the imperative to save lives that might otherwise be lost to the tyranny of distance and time.

## Data Availability

The original contributions presented in the study are included in the article/Supplementary Material, further inquiries can be directed to the corresponding authors.
