# Supplementary material for: Mapping the research landscape of UAV- and eVTOL-enabled emergency medical services: a bibliometric analysis, scoping review, and policy tool assessment
Source: Front Health Serv. 2026 Oct 15;6:1861375. doi: 10.3389/frhs.2026.1861375 (PMC13716796; doi:10.3389/frhs.2026.1861375)
Supplement: Supplementary file 1 [file Table1.docx]

**Supplementary Material**

**Mapping the Research Landscape of UAV- and eVTOL-Enabled Emergency Medical Services: A Bibliometric Analysis, Scoping Review, and Policy Tool Assessment**

Manuscript ID 1861375. Frontiers in Public Health.

**Supplementary Material S1. Complete Database-Specific Search Strategies**

The strategies below operationalize, in each database's native syntax, the two concept blocks reported in Section 2.2.1 of the manuscript: (a) aerial platform terms and (b) emergency medical terms, connected by Boolean AND. Searches covered publications from 1 January 2015 through 31 March 2026, with the final update completed in March 2026. No language restrictions were applied, and no document-type restrictions were applied at the search stage; conference abstracts without full-text access, editorials and commentaries without original data, and other ineligible record types were excluded during screening according to the criteria in Section 2.3.1. All records were exported in RIS format for deduplication and screening.

**S1.1 Web of Science Core Collection (WoSCC)**

Citation indexes: Science Citation Index Expanded (SCIE), Social Sciences Citation Index (SSCI), and Emerging Sources Citation Index (ESCI). Search field: Topic (TS), which covers title, abstract, author keywords, and Keywords Plus. Timespan: 2015-01-01 to 2026-03-31. Records retrieved: 423.

```
TS=(("eVTOL" OR "electric vertical takeoff" OR "electric vertical take-off"
  OR "air taxi" OR "UAV" OR "unmanned aerial vehicle" OR "drone"
  OR "advanced air mobility" OR "urban air mobility")
AND
("emergency medical" OR "EMS" OR "ambulance" OR "cardiac arrest" OR "AED"
  OR "defibrillator" OR "medical rescue" OR "blood delivery"
  OR "organ transport" OR "prehospital" OR "pre-hospital" OR "HEMS"
  OR "air ambulance"))
```

**S1.2 Scopus**

Search field: TITLE-ABS-KEY. Date limit applied through the publication-year facet and the March 2026 final update. Records retrieved: 582.

```
TITLE-ABS-KEY(("eVTOL" OR "electric vertical takeoff" OR
  "electric vertical take-off" OR "air taxi" OR "UAV" OR
  "unmanned aerial vehicle" OR "drone" OR "advanced air mobility" OR
  "urban air mobility")
AND
("emergency medical" OR "EMS" OR "ambulance" OR "cardiac arrest" OR
  "AED" OR "defibrillator" OR "medical rescue" OR "blood delivery" OR
  "organ transport" OR "prehospital" OR "pre-hospital" OR "HEMS" OR
  "air ambulance"))
AND PUBYEAR > 2014
```

**S1.3 PubMed/MEDLINE (benchmark series)**

As stated in Section 2.2.1, PubMed/MEDLINE was queried with an equivalent strategy but its records were **not merged into the bibliometric corpus**, which derives exclusively from WoSCC, Scopus, CNKI, and Wanfang after deduplication. PubMed records served as an independently indexed, MeSH-curated benchmark series for the temporal trend analysis in Section 3.1.1 and Figure 2. Search fields: Title/Abstract. Date filter: 2015/01/01 to 2026/03/31.

```
("eVTOL"[Title/Abstract] OR "electric vertical takeoff"[Title/Abstract]
  OR "electric vertical take-off"[Title/Abstract]
  OR "air taxi"[Title/Abstract] OR "UAV"[Title/Abstract]
  OR "unmanned aerial vehicle"[Title/Abstract] OR "drone"[Title/Abstract]
  OR "advanced air mobility"[Title/Abstract]
  OR "urban air mobility"[Title/Abstract])
AND
("emergency medical"[Title/Abstract] OR "EMS"[Title/Abstract]
  OR "ambulance"[Title/Abstract] OR "cardiac arrest"[Title/Abstract]
  OR "AED"[Title/Abstract] OR "defibrillator"[Title/Abstract]
  OR "medical rescue"[Title/Abstract] OR "blood delivery"[Title/Abstract]
  OR "organ transport"[Title/Abstract] OR "prehospital"[Title/Abstract]
  OR "pre-hospital"[Title/Abstract] OR "HEMS"[Title/Abstract]
  OR "air ambulance"[Title/Abstract])
AND ("2015/01/01"[Date - Publication] : "2026/03/31"[Date - Publication])
```

**S1.4 CNKI (China National Knowledge Infrastructure)**

Search field: subject (主题, SU), professional search mode. Date limit: 2015-01-01 to 2026-03-31. CNKI and Wanfang jointly contributed 145 records after within-source deduplication.

```
SU=('低空经济'+'无人机'+'电动垂直起降'+'eVTOL'+'空中出租车'+'城市空中交通'
    +'先进空中交通')
AND
SU=('紧急医学救援'+'急救'+'院前急救'+'心脏骤停'+'自动体外除颤器'+'除颤仪'
    +'血液配送'+'器官转运'+'航空医疗救援'+'空中救护'+'医疗救援')
```

**S1.5 Wanfang Data**

Search field: subject (主题). Date limit: 2015 to March 2026. See S1.4 for the combined CNKI/Wanfang record count.

```
主题:(低空经济 OR 无人机 OR 电动垂直起降 OR eVTOL OR 空中出租车 OR 城市空中交通)
AND
主题:(紧急医学救援 OR 急救 OR 院前急救 OR 心脏骤停 OR 自动体外除颤器 OR 除颤仪
      OR 血液配送 OR 器官转运 OR 航空医疗救援 OR 空中救护 OR 医疗救援)
```

**S1.6 Supplementary Identification**

Reference lists of included reviews and of prior landscape mappings were scanned manually, contributing 35 additional records. In total, 1,185 records were identified (database searching 1,150; reference list scanning 35).

**S1.7 Deduplication and Record Harmonization**

All records were exported in RIS format and imported into reference-management software. Duplicates were removed in two passes: exact DOI matching, followed by normalized title-author-year matching (case-folding, punctuation and whitespace normalization); residual near-duplicates, including translated duplicate records of the same study, were verified and resolved manually. This procedure removed 502 duplicates, leaving 683 unique records for title and abstract screening; all 683 were retained for the bibliometric analysis. Chinese-language records were harmonized with the international set prior to analysis by translating title and keyword fields into English and standardizing institutional name variants (for example, unifying transliterated and translated forms of the same institution), so that keyword co-occurrence and institutional collaboration analyses treat each entity consistently.

**Supplementary Table S2. Study-Level Charting of Included Studies (n = 168)**

The table applies the data charting framework described in Section 2.3.2. Domain codes: D1, drone AED delivery for out-of-hospital cardiac arrest; D2, medical supply and biological specimen transport; D3, eVTOL patient transport and air ambulance services (rows marked "D3 context" chart traditional HEMS evidence synthesized as comparator context in Section 3.2.4); D4, health system integration and policy. Bracketed numbers refer to the reference list of the revised manuscript. Rows 1-20 chart the exemplar studies foregrounded in Sections 3.2.2 through 3.2.5. Rows 21-168 were reconstructed through a re-executed, item-verified search following the strategies in S1 (working version); bracketed reference numbers for these rows will be assigned when the manuscript reference list is extended at final lock-in. Verification tags at the end of each Key finding(s) cell: [A] verified directly against authoritative records; [B]/[B-PDF] verified via peer-reviewed reference lists (B-PDF: from the two open-access reviews provided by the authors); [C] complete bibliographic records pending final one-by-one confirmation. Rows 77-84 were completed from the authors’ CNKI/Wanfang exports and unified to the common row architecture; rows flagged “for author review” or “for author confirmation” carry a domain-assignment or eligibility note to resolve during final screening. All verification tags are working annotations to be removed once the table is locked.

| **No.** | **Study [Ref.]** | **Country/region** | **Design** | **Platform** | **Domain** | **Sample/scale** | **Key finding(s)** |
| --- | --- | --- | --- | --- | --- | --- | --- |
| 1 | Schierbeck 2023 [7] | Sweden (Gothenburg region) | Prospective observational deployment | Multirotor AED-delivery drone | D1 | 55 suspected OHCA cases | Drones arrived before ambulances in 67% of cases; median time advantage 3 min 14 s. |
| 2 | Schierbeck 2022 [29] | Sweden | Case report | Drone-delivered AED | D1 | 1 OHCA case | First reported real-world use of a drone-delivered AED in OHCA before ambulance arrival. |
| 3 | Schierbeck 2026 [33] | Sweden | Prospective operational study with dispatcher educational bundle | Drone-delivered AED, dispatcher referral | D1 | Consecutive drone-eligible suspected OHCA alerts | AED attachment rate rose from 27% to 50% after a structured dispatcher educational bundle. |
| 4 | Jakobsen 2025 [8] | Denmark (Aalborg) | Prospective feasibility study | Semi-autonomous AED-delivery drone | D1 | 16 eligible cases | 100% delivery success, no adverse events; median drone response 4 min 47 s vs ambulance 3 min 25 s, highlighting base-placement dependence. |
| 5 | Jakobsen 2024 [18] | International | Scoping review | Drone AED delivery | D1 | Published drone-AED literature | Maps feasibility, dispatch integration, and outcome evidence for drone-delivered AEDs in OHCA. |
| 6 | Boutilier 2017 [32] | Canada (Toronto region) | Combined optimization-queuing network model | Drone network | D1 | 53,702 historical cardiac arrests | Mathematically designed drone networks systematically shorten AED arrival relative to historical response. |
| 7 | Maaz 2025 [9] | Canada (Ontario) | Markov microsimulation, cost-effectiveness analysis | Drone networks (964 configurations) | D1 | 22,017 OHCAs over a decade | All configurations cost-effective under model assumptions; smallest non-dominated network (20 drones) ICER $20,912/QALY; response time reduced 32-71%; survival to discharge improved 21-46%. |
| 8 | Qin 2025 [34] | China (Xuzhou) | GIS-based network optimization | AED/drone deployment siting | D1 | City-scale deployment analysis | Optimizes AED drone deployment within the walking golden window; peer-reviewed Chinese deployment evidence at an early stage. |
| 9 | Nisingizwe 2022 [10] | Rwanda | Retrospective operational analysis | Fixed-wing delivery drone (Zipline) | D2 | 12,733 blood product deliveries | 61% reduction in blood delivery time; 67% decrease in blood unit expiration. |
| 10 | Heidet 2023 [11] | France (Greater Paris); Canada (Metro Vancouver) | International simulation-based cohort | VTOL air ambulance (1-2 units) | D3 | 6,217 real OHCA cases (modeled) | ALS arrival reduced from 21 to 7-9 min (Paris) and 12 to 7-9 min (Vancouver); significant across evaluated metrics. |
| 11 | Goyal 2022 [35] | United States | Multi-criteria feasibility analysis | eVTOL air ambulance | D3 | Operational and technology parameters | Battery technology the primary constraint; approximately four-fold charge-time reduction needed to compete with ground ambulances below 40 km. |
| 12 | Galvagno 2012 [30] | United States | Retrospective registry cohort | Helicopter EMS | D3 context | Adults with major trauma (national registry) | HEMS associated with improved survival to discharge relative to ground EMS for adults with major trauma. |
| 13 | Orso 2025 [31] | International | Systematic review, meta-analysis, meta-regression, trial sequential analysis | Helicopter EMS | D3 context | 77 studies, approximately 2.6 million patients | No demonstrable survival benefit vs ground EMS (RR 1.13, 95% CI 0.96-1.34; very low quality); total incremental net benefit approximately EUR 980,000 per QALY. |
| 14 | Fritz 2024 [36] | International | Systematic review and meta-analysis (ISS >= 9) | Helicopter scene response | D3 context | Injured patients with ISS of at least nine | Survival benefit for helicopter scene response in this subgroup, complicating all-comer null findings. |
| 15 | De Silvestri 2023 [16] | Europe/international | Scoping review | Healthcare drones | D4 | Healthcare-drone challenge literature | Only one included study substantively addressed regulatory frameworks, limited to Polish and EU law. |
| 16 | Zhang 2026 [17] | International | Review | UAVs in prehospital care | D4 | Prehospital UAV literature | Identifies regulatory uncertainty, airspace integration, public acceptance, and workforce readiness as principal barriers. |
| 17 | Zhu 2026 [3] | China | Systems exploration and policy analysis | eVTOL emergency medical rescue system | D4 | National system design, scenario classes | First systematic exploration of eVTOL-enabled emergency medical rescue within the Fifteenth Five-Year Plan framework, covering high-rise, mountainous, subterranean, and open-water scenarios. |
| 18 | Bao 2024 [37] | China | Perspective, system-construction proposal | Aviation medical rescue system | D4 | National proposal | Proposals for accelerating construction of an aviation medical emergency rescue system. |
| 19 | Li 2025 [38] | China (Qingyuan County) | Practice report | County-level air rescue system | D4 | County practice experience | Documents county-level air emergency rescue practice in the mountainous southwest. |
| 20 | Chen 2025 [39] | China (remote areas) | Comparative evaluation | Air-ground integrated emergency system | D4 | Critically ill patients in remote areas | Air-ground integrated response evaluated for the treatment of critically ill patients in remote areas. |
| 21 | Rosamond 2020 | United States (North Carolina) | Randomized simulation, drone vs ground AED search | Multirotor AED-delivery drone | D1 | 35 simulated OHCA events | Drone delivery faster than ground AED search in 4 of 5 zones (mean 4:45 vs 6:06 min). [A] |
| 22 | Claesson 2017 | Sweden (Norrtalje) | Test flights vs historical EMS times | Multirotor AED-delivery drone | D1 | 18 BVLOS test flights | Drone arrived before EMS in all 18 flights; median time reduction 16:39 min. [A] |
| 23 | Claesson 2016 | Sweden (Stockholm County) | GIS modeling with delivery test flights | Multirotor drone; latch, landing, parachute release | D1 | 3,165 historical OHCAs; 13 test flights | Incidence-balanced drone bases reached 32% of urban and 93% of rural OHCAs before EMS. [A] |
| 24 | Schierbeck 2021 | Sweden | GIS coverage modeling | AED-equipped drone network | D1 | 39,246 registry OHCAs | Covering 50/80/90/100% of OHCAs within 8 min requires 21/366/784/2,408 drone systems. [A] |
| 25 | Cheskes 2020 | Canada (Ontario) | Drone delivery feasibility simulation | Multirotor AED-delivery drone | D1 | 6 simulated rural OHCAs | AED arrived 1.8-8.0 min before ambulance in all flights. [A] |
| 26 | Leung 2022 | Canada (British Columbia) | Base-location optimization modeling | AED-delivery drone network | D1 | Historical OHCA cohort; 5 base configurations | All configurations reduced median AED arrival; drones preceded first responders in 53-77% of cases. [A] |
| 27 | Chu 2021 | Canada (Ontario, Peel) | Machine-learning dispatch modeling | AED-delivery drone network | D1 | 3,573 historical OHCAs | ML dispatch rules cut drone dispatches about 30% while preserving response-time gains. [A] |
| 28 | Derkenne 2021 | France (Greater Paris) | Real-world-based simulation | AED-delivery drone | D1 | 3,014 registry OHCAs | Drone AED preceded BLS teams in 26% of cases, with mean gain 190 +/- 7 s in 93% of those. [A] |
| 29 | Baumgarten 2022 | Germany (rural Vorpommern-Greifswald) | Simulation with bystander interviews | BVLOS AED-delivery drone | D1 | 46 simulated OHCAs | Chain-of-survival integration feasible and safe; 93.8% flight reliability. [A] |
| 30 | Rees 2021 | United Kingdom (Wales) | Simulation proof-of-concept | Fixed-wing drone, parachute AED drop | D1 | 6 BVLOS flights | 4 of 6 flights delivered the AED by parachute (about 50 m precision). [A] |
| 31 | Fischer 2023 | Austria (mountainous region) | Simulation feasibility study | BVLOS drone with long-line AED release | D1 | 29 simulated mountain OHCAs | Mountain AED delivery feasible; 93% of participants felt safe at drone approach. [A] |
| 32 | Sanfridsson 2019 | Sweden | Mixed-methods simulation | AED-delivery drone | D1 | 8 simulated OHCAs, older lay bystanders | Bystanders felt relief rather than fear; difficulties centred on phone use and electrode placement. [A] |
| 33 | Bauer 2021 | Germany | Comparative economic modeling | UAV networks for early defibrillation | D1 | 1,427 modeled cases across 329 counties | Full coverage needs 1,933 UAVs; 90% coverage ICER 14,548 EUR per life-year, cost-effective. [A] |
| 34 | Roeper 2023 | Germany (rural district) | Economic evaluation | AED-delivery drone network | D1 | District-level network comparison | Drone network more cost-efficient than building and maintaining a stationary AED network. [A] |
| 35 | Liu 2023 | China (Shandong) | Review | AED-delivery drones | D1 | Published drone-AED literature | Positions drone-delivered AEDs as a strategy to improve OHCA prognosis. [A] |
| 36 | Mermiri 2020 | Greece | Review | AED-delivery drones | D1 | Published drone-AED literature | Reviews the state of drone AED delivery research; volume/pages to confirm. [B] |
| 37 | Mackle 2020 | United Kingdom (Northern Ireland) | Genetic-algorithm simulation | Aerial ambulance drone network | D1 | 78-site modeled network | Mean response times cut about 25% in Belfast and 50% elsewhere vs ambulance or bystander retrieval. [A] |
| 38 | Smith 2025 (Paramedicine) | United Kingdom (Wales) | Simulation feasibility study | BVLOS AED-delivery drone | D1 | 11 simulations, 9 completed flights | 81.8% delivery success; human-drone interaction was the chief delay source. [A] |
| 39 | Starks 2024 (JACC Adv) | United States (North Carolina) | Logistic-regression network modeling | First-responder AEDs plus drone network | D1 | 28,292 historical OHCAs, 48 counties | AED arrival under 5 min rose from 16.5% to 56.3%; predicted witnessed-OHCA survival 14.5% to 19.4%. [A] |
| 40 | Starks 2024 (Circ Cardiovasc Qual Outcomes) | United States (North Carolina) | Public simulation with telecommunicator CPR | Drone-delivered AED | D1 | 51 single-bystander simulations | Timed bystander AED retrieval and CPR quality inform 5-minute deployment targets. [A] |
| 41 | Sedig 2020 | Canada (Ontario, Caledon) | Qualitative study, interviews and focus groups | AED-delivery drone program | D1 | 65 community residents | Concept broadly acceptable; community CPR and AED literacy the decisive readiness factor. [B] |
| 42 | Schierbeck 2022 (Eur Heart J) | Sweden (Gothenburg) | Real-world feasibility study | AED drones integrated with EMS dispatch | D1 | 12 real suspected-OHCA dispatches; 61 BVLOS test flights | 11 of 12 deliveries successful; drone first in 64% (median gain 1:52); 90% test-flight reliability. Final volume, pages and DOI to be confirmed at OUP. [B-PDF/C] |
| 43 | Lim 2022 | Singapore | Scoping review | Drones in OHCA | D1 | 26 included studies | Maps time savings, optimisation models and implementation barriers for AED drones. [A] |
| 44 | Leith 2024 | United States (Michigan) | Randomized simulation, qualitative arm | Grounded multipurpose medical drone | D1 | 24 simulated emergencies | Lay bystanders interacted effectively and comfortably with the drone. [B-PDF] |
| 45 | Yukun 2023 | China | Improved immune-algorithm siting model | First-aid AED drone network | D1 | 300 simulated OHCA sites | Improved algorithm raised system stability about 29%; estimated response 2:07 min. [B-PDF] |
| 46 | van Veelen 2024 | Italy (alpine park) | Randomized simulation trial | AED-delivery drone | D1 | Park-visitor simulations | Call-to-shock 2.2 min with drone vs 12.4 min with bystander AED retrieval; author list/DOI to complete. [B] |
| 47 | Srivilaithon 2025 | Thailand (suburban campus) | Feasibility test flights | DJI M600 with 2 kg vs 4 kg AED payloads | D1 | 90 test flights | 97.7% success; payload mass shaped speed and battery trade-offs. [A] |
| 48 | Pi 2025 | China | Simulation time-efficiency comparison | AED-delivery drone | D1 | Simulated OHCA scenarios | Compares drone vs conventional AED access time efficiency; DOI to confirm. [A] |
| 49 | Ryan 2021 | United States (Virginia) | GIS feasibility modeling | Medical UAS network | D1 | 18 modeled sites | EMS-base plus post-office siting raised under-5-min coverage from 29.7% to 70.1%. [A] |
| 50 | Pulver 2016 | United States (Utah) | Maximum-coverage siting optimization | AED-enabled medical drones | D1 | County-scale demand model | One-minute AED arrival coverage rose from 4.3% (EMS) to 90.3% with optimized sites. [B-PDF] |
| 51 | Pulver 2018 | United States (Utah) | Backup-coverage siting optimization | Medical drone network | D1 | County-scale model | Backup-coverage weighting trades primary coverage for redundancy (56-75 drones). [B-PDF] |
| 52 | Wankmueller 2024 | Austria (mountainous region) | Simulation and challenge analysis | AED-delivery drones | D1 | Mountain-region scenarios | Details potential and operational challenges of airborne mountain AED delivery. [A] |
| 53 | Nenni 2020 | Italy | System design study | Drone-supported EMS | D1 | Conceptual system model | Develops a drone-supported EMS design with operational parameters. [B] |
| 54 | Bogle 2019 | United States (North Carolina) | Statewide optimization with cost-effectiveness | AED drone network | D1 | 16,503 historical OHCAs | 500-drone network: median AED arrival 7.7 to 2.7 min; survival 12.3% to 24.5%; about USD 858 per QALY. [A] |
| 55 | Zegre-Hemsey 2018 | United States | Review and perspective | AED-delivery drones | D1 | Published literature | Frames implications of drone AED delivery for emergency cardiac care. [B] |
| 56 | Zegre-Hemsey 2020 | United States (North Carolina) | Qualitative simulation study | Drone-delivered AED | D1 | 17 interviewed participants of 35 simulations | User concerns centred on CPR and defibrillation rather than the drone itself. [A] |
| 57 | Zegre-Hemsey 2024 | United States/Sweden/Canada | Comparative qualitative analysis | Drone AED programs | D1 | Three national programs | Real-time integration challenges: regulation, dispatch and human factors. [A] |
| 58 | Ni 2024 | China | Review, Chinese literature | Emergency rescue drones | D1 | Published literature | Reviews drone applications supporting out-of-hospital CPR. [A] |
| 59 | Scholz 2023 | Germany (rural) | Night-flight feasibility study | BVLOS AED-delivery UAS | D1 | 10 night and 10 day flights | Night-time AED delivery feasible with no relevant operational differences from daytime. [B-PDF] |
| 60 | Purahong 2022 | Thailand | System development and field test | Medical drone with bystander app | D1 | Operational field tests | Smartphone-app-triggered AED drone dispatch produced successful deliveries. [B-PDF] |
| 61 | Kim 2021 | South Korea | Mixed-methods simulation | Drone-delivered AED with dispatcher CPR | D1 | 24 simulated OHCAs | Video versus audio dispatcher instructions improved CPR quality; drone delivery valued. [B-PDF] |
| 62 | Davidson 2024 | United States (Michigan) | Randomized simulation pilot | Grounded medical drone | D1 | 24 simulated emergencies | Drone-specific dispatch instructions improved safety and efficacy of bystander interaction. [B-PDF] |
| 63 | Ren 2024 | China | Vertiport location modeling | Medical drone vertiports | D1 | 62 simulated OHCA sites | 24 launch sites ensure 4-min AED arrival; estimated survival benefit up to 64%. [B-PDF] |
| 64 | Frigstad 2023 | Sweden | Joint optimization modeling | Drones plus volunteer responders | D1 | 20 modeled launch sites | Joint dispatch cut AED arrival by 5:29 min; estimated survival 9.6% to 15.8%. [B-PDF] |
| 65 | Lancaster 2021 (Resusc Plus) | United States (Washington) | Computer simulation | Novel cardiac arrest response systems | D1 | 10-year simulated horizon | Maps cost-survival trade-offs; drone AED systems cost-effective at scale. [B-PDF] |
| 66 | Lancaster 2021 (IISE Trans Healthc Syst Eng) | United States (Washington) | Monte Carlo simulation | Mobile responders plus five AED drones | D1 | City-scale simulation, Bellevue | Earlier defibrillation raised estimated survival from 20% to 30%. [B-PDF] |
| 67 | Wankmueller 2020 | Austria (mountainous region) | Facility-location optimization | Defibrillator drones, two speed classes | D1 | 36 modeled launch sites | Fast drones from 36 sites beat air-ambulance response times in mountain terrain. [B-PDF] |
| 68 | Choi 2021 | South Korea (Seoul) | Topography-weather flight simulation | UAV-AED, basic vs advanced models | D1 | 18,856 registry OHCAs | Topographic routing added about 1.6 min vs Euclidean paths; weather rules did not reduce call-to-attach time. [B-PDF] |
| 69 | Van de Voorde 2017 | Belgium/Netherlands | Short report with simulation data | Drone-ambulance AED concept | D1 | Concept demonstration | Positions the AED drone as a third-generation defibrillation access route. [C] |
| 70 | Sanz-Martos 2022 | Spain | Systematic review | Drones for emergency and urgent care | D1 | Published literature | Synthesizes emergency-care drone applications including AED delivery; details at final check. [C] |
| 71 | Boutilier 2022 | Canada (Toronto region) | Integrated location-queuing optimization | AED drone network design | D1 | Regional network model | Integrated model needs fewer bases and drones than region-specific designs for equal gains. [C] |
| 72 | Rachunok 2016 | United States (North Carolina) | Dispatch simulation, conference full paper | UAV AED response | D1 | 168 candidate UAV sites | Modeled survival probability and response-time gains under dispatch rules; retain at authors' discretion. [B-PDF] |
| 73 | Glick 2020 | United States (Oregon) | Reliability modeling, conference full paper | Time-sensitive drone AED delivery | D1 | City-scale reliability model | Quantified delivery failure rates from range and meteorological constraints; retain at authors' discretion. [B-PDF] |
| 74 | Smith 2025 (Resusc Plus) | United Kingdom | End-to-end integration simulation | BVLOS AED drone linked to 999 call flow | D1 | Simulated OHCAs, controlled site | Call-to-takeoff 2.18 min; autonomous flight with live call-handler communication; volume to confirm. [A] |
| 75 | Bernstein 2025 | United Kingdom | Qualitative interview study | Drone-delivered AEDs | D1 | 14 real-life OHCA bystanders | TDF/COM-B mapping of barriers and facilitators to retrieving and using drone AEDs. [A] |
| 76 | Jiang 2025/2026 | China (Kunming) | Pilot integration study | Wearable ECG vest plus AED drone, high-rise | D1 | One full-chain simulation | AED delivered 7:14 after simulated arrest, 6:49 faster than ambulance; authors, journal and date to complete via PMC; window boundary check. [A] |
| 77 | Yu 2021 | China | Review (Chinese literature) | Emergency-rescue UAVs | D1 | Published literature | Reviews UAV applications and development in China's emergency rescue, including medical scenarios; domain assignment (D1 vs D4) for author review. [B] |
| 78 | CSEM Consensus 2020 | China | Expert consensus statement | AED deployment, incl. drone delivery | D1 | National consensus panel | National expert consensus on AED layout and deployment; drone-based delivery treated as an emerging mode; drone-content share for author confirmation. [B] |
| 79 | Suo 2026 | China (urban) | Feasibility and time-efficiency study | TD9 rappelling hexacopter | D1 | 43 flights | Alert-to-AED-release 353 s vs EMS arrival 597 s (cross-matched with Xiao 2026 charting); early-2026 issue, within window. [B] |
| 80 | Wei 2025 | China | Review (Chinese literature) | AED-delivery drones | D1 | Published literature | Reviews AED-drone applications in emergency rescue. [B] |
| 81 | Chen 2025 (China Storage & Transport) | China | Integrated delivery-path analysis | Multi-tier AED deployment incl. drones | D1 | Layout-scheme analysis | Analyzes rapid-delivery routing for a three-dimensional AED layout; trade-journal source, retain at authors' discretion. [B] |
| 82 | Jie 2025 | China | Multi-objective deep reinforcement-learning siting model | Fixed, mobile and drone collaborative AED delivery | D1 | City-scale siting instances | Deep-RL model solves the collaborative fixed-mobile-drone AED siting problem. [B] |
| 83 | Xu 2026 | China | Demonstration-project practice report | Road-air integrated medical delivery system | D1 | Demonstration project | Practice and exploration of a road-air integrated medical emergency delivery demonstration; domain assignment (D1 vs D2) for author review; early-2026 issue, window check. [B] |
| 84 | Liu 2024 (Chin J Emerg Med) | China | Review | AI decision support for OHCA, incl. drone-AED dispatch | D1 | Published literature | Reviews AI decision support across the OHCA response chain; drone-dispatch content share and eligibility for author confirmation. [B] |
| 85 | Amukele 2015 | United States | Laboratory comparison study | Small UAS specimen transport | D2 | Paired routine laboratory specimens | Drone transport did not alter routine chemistry, hematology or coagulation results. [A] |
| 86 | Amukele 2016 | United States | Laboratory microbiology study | Drone specimen transport | D2 | Blood and sputum culture specimens | Transport did not affect microbial recovery or culture results. [A] |
| 87 | Amukele 2017 (Am J Clin Pathol) | United States | Long-distance transport study | Drone, extended-duration flight | D2 | Paired samples over a long flight | Most analytes concordant; temperature-sensitive analytes flag cold-chain control needs. [A] |
| 88 | Amukele 2017 (Transfusion) | United States | Blood-product transport study | Multirotor drone | D2 | RBC, platelet and plasma units | No adverse effects on red cell, platelet or plasma quality markers after flight. [A] |
| 89 | Homier 2021 | Canada (Montreal) | Prospective pilot, drone vs ground | Blood-product delivery drone | D2 | Simulated blood products to urban trauma centre | Urban drone delivery feasible against ground-courier benchmarks. [A] |
| 90 | Yakushiji 2020 | Japan | Evidential quality study | Transfusion-material drone | D2 | Transported blood samples | Blood quality unaffected by drone transport. [B] |
| 91 | Yakushiji 2021 | Japan (remote island) | Quality-control transport study | Drone flights to a remote island | D2 | RBC solutions for transfusion | RBC quality maintained across island drone flights; volume/issue pending. [B] |
| 92 | Bates 2021 | United Kingdom | Experimental parachute-delivery study | Parachute airdrop of blood | D2 | RBC and plasma units | Parachute delivery did not degrade RBC or plasma quality measures. [B] |
| 93 | Lammers 2023 | United States | Review, combat logistics | UAV blood and medical logistics | D2 | Military use-case synthesis | Frames UAV blood delivery for prolonged field care and combat zones; pages pending. [A] |
| 94 | [Authors pending] 2024 (Br J Haematol) | To confirm | Prospective randomised controlled laboratory study | Drone transport of blood components | D2 | Blood component units, flight vs control | Randomised laboratory evidence on drone effects on component quality; complete via Wiley record. [A] |
| 95 | Scalea 2018 | United States (Maryland) | Experimental organ-transport study | UAS with real-time organ-status sensor | D2 | Human kidney test flights | Organ transport with live monitoring feasible; vibration below fixed-wing benchmarks. [A] |
| 96 | Scalea 2019 | United States | Feasibility and validation study | Organ-transport drone | D2 | Kidney flights with biopsy assessment | No histologic damage after drone flights; supports organ UAS transport. [A] |
| 97 | Scalea 2021 | United States (Baltimore) | Clinical implementation report | Custom organ-delivery UAS | D2 | One human kidney delivered | First UAS delivery of a human organ for successful transplantation. [B] |
| 98 | Sage 2022 | Canada (Toronto) | Real-world technical study | Organ-transport drone | D2 | Human lung delivery between hospitals | Real-world drone transport of a human lung for transplantation validated. [B] |
| 99 | Ornato 2020 | United States (Virginia) | Simulation feasibility study | Naloxone-delivery drone | D2 | 30 bystander simulations | Bystanders gave drone-delivered naloxone under dispatcher guidance; contact-to-dose about 122 s. [A] |
| 100 | Thiels 2015 | United States | Feasibility and concept analysis | Medical-product transport UAV | D2 | Use-case and regulatory analysis | Early framework for UAV transport of blood products and pharmaceuticals. [A] |
| 101 | Kremer 2025 | Ghana (Ashanti) | Mixed-methods impact assessment | Fixed-wing delivery network (Zipline) | D2 | Regional maternal-health deliveries | Aerial logistics improved maternal-health and emergency supply outcomes. [A] |
| 102 | Flemons 2022 | Canada (remote First Nations) | Implementation study | Delivery drone, COVID-19 kits and supplies | D2 | Remote-community deliveries | Drone delivery of diagnostic kits and supplies feasible during COVID-19. [A] |
| 103 | Aggarwal 2023 | India (North-East) | Implementation experience study | Medical-supply delivery drones | D2 | Program deliveries | Documents experiences, challenges and adopted strategies for drone delivery. [A] |
| 104 | Naor 2024 | Israel | Case study, Industry 5.0 | VTOL parcel delivery to hospitals | D2 | Hospital logistics case | VTOL distribution to hospitals analysed within an Industry 5.0 frame. [A] |
| 105 | Euchi 2021 | Tunisia | Analytical review | Pandemic medical-supply drones | D2 | Healthcare logistics problem space | Assesses realistic roles and limits of drones for pandemic supply chains. [A] |
| 106 | Ling 2019 | United States | Review with program data | Blood-delivery drones | D2 | Field-program synthesis | Surveys aerial blood delivery feasibility; issue and pages pending Wiley check. [B] |
| 107 | Mesar 2018 | United States | Short report | Supply drone for prolonged field care | D2 | Field-care demonstration | Drone resupply during prolonged field care feasible. [B] |
| 108 | Li 2023 | China | System design and demonstration | Emergency drug-supply UAV system | D2 | System-level design | Designs a drone-based emergency medical materiel support system. [A] |
| 109 | Zhu 2026 (Front Public Health) | China | Multi-objective optimization modeling | Urban medical-delivery drones | D2 | Dynamic airspace allocation model | Optimizes dynamic airspace allocation for urban medical emergency delivery; window boundary check. [A] |
| 110 | Haidari 2016 | Mozambique (modeled LMIC) | Supply-chain simulation (HERMES) | Vaccine-delivery UAS | D2 | National-scale vaccine chain model | UAS improved vaccine availability and lowered logistics cost per dose vs land transport. [C] |
| 111 | Mateen 2020 | Zambia (modeled) | Network modeling case study | Antiepileptic-drug delivery drones | D2 | National delivery framework | Framework and modeling for drone drug distribution in a low-income country. [C] |
| 112 | Hii 2019 | United Kingdom | Pharmaceutical stability study | Medicine-delivery drone | D2 | Insulin transport tests | Drone flight maintained insulin quality within accepted limits. [C] |
| 113 | Knoblauch 2019 | Madagascar, Malawi, Senegal | Implementation field study | Bi-directional medical drones | D2 | Multi-country field operations | Lessons for integrating bi-directional transport of samples and supplies. [C] |
| 114 | Eichleay 2019 | Low- and middle-income settings | Decision-tool development | Medical-supply UAV | D2 | UAV Delivery Decision Tool | Structured tool for deciding when drone delivery adds value. [C] |
| 115 | Poljak 2020 | Slovenia | Review | Drones in clinical microbiology and ID | D2 | Published literature | Status, challenges and barriers for microbiology and infectious-disease drone use. [C] |
| 116 | Oakey 2021 | United Kingdom | Vibration quantification study | Fixed-wing and multicopter drones | D2 | Medicine payload vibration tests | Characterizes vibration exposure of medicines in transit by drone type. [C] |
| 117 | Johannessen 2021 | Norway | Logistics modeling | Laboratory-sample drone network | D2 | University-hospital laboratory volume | A drone model can carry a large laboratory's full analytic volume with time savings. [C] |
| 118 | Kim 2017 | United States | Operations-research modeling | Rural chronic-care drones | D2 | Rural service network model | Drone-aided delivery framework for chronic-disease care in rural areas. [C] |
| 119 | Sham 2022 | Malaysia | Cross-sectional attitude survey | Medicine and vaccine delivery drones | D2 | Rural health-care workers | Health-worker attitudes toward pandemic drone delivery in rural centres. [C] |
| 120 | Comtet 2021 | Norway | Cross-sectional survey study | Healthcare drone adoption | D2 | Health-system staff sample | Pro-innovative leadership and gender moderate readiness for drone transport. [C] |
| 121 | Johannessen 2022 | Norway | Conceptual cost-competitiveness model | Biologic-sample drones | D2 | Time and cost assessment framework | Framework for time savings and cost competitiveness of sample drones. [C] |
| 122 | Espejo-Diaz 2025 | Colombia | Operations-research optimization | AAM/eVTOL vehicles for EMS | D3 | Vertiport siting model | AAM vertiport siting improves EMS access for hard-to-reach areas. [A] |
| 123 | Mihara 2021 | Japan | Cost modeling, conference full paper | eVTOL air ambulance | D3 | Configuration cost analysis | Battery-swap costs dominate; near-term eVTOL cost benefit for air EMS is limited. [A] |
| 124 | de Souza 2025 | Brazil (Amazon) | GIS spatial assessment | eVTOL (Joby S4) vs helicopter (H125) | D3 | Regional coverage comparison | eVTOL viable as complementary remote-area emergency transport. [A] |
| 125 | Bridgelall 2024 | United States (North Dakota) | GIS optimization | AAM for rural healthcare logistics | D3 | Statewide siting model | Vertiport siting achieves about 22-min mean rural reach for medical missions. [A] |
| 126 | Peng 2022 | China | Bibliometric analysis (CiteSpace) | Helicopter EMS literature | D3 context | 1,378 records | Maps HEMS research hotspots including HEMS-vs-ground comparisons. [A] |
| 127 | Brown 2016 (Surgery) | United States | Retrospective NTDB cohort, propensity-adjusted | Helicopter vs ground EMS | D3 context | National trauma registry adults | HEMS survival benefit persisted even without a time-saving advantage. [A] |
| 128 | Brown 2016 (J Trauma Acute Care Surg) | United States | Retrospective pediatric NTDB cohort | Helicopter scene transport | D3 context | Pediatric trauma, 2007-2012 | Scene HEMS associated with improved pediatric survival. [A] |
| 129 | Blaesius 2021 | Germany | Matched registry analysis (TraumaRegister DGU) | Helicopter vs ground EMS | D3 context | 2,755 pediatric patients | HEMS survival benefit, OR 0.489 (95% CI 0.282-0.850). [A] |
| 130 | Tsuchiya 2016 | Japan | Nationwide cohort, propensity and instrumental variable | Helicopter vs ground EMS | D3 context | 21,286 JTDB patients | Lower in-hospital mortality with HEMS (matched 22.2% vs 24.5%). [A] |
| 131 | Alstrup 2021 | Denmark | Nationwide cohort study | Helicopter vs ground transport | D3 context | 10,618 patients | No adjusted 1-year mortality difference (23.2% vs 24.5%, HR 0.94). [A] |
| 132 | Wu 2020 | Taiwan, China (Penghu) | Retrospective descriptive study | Helicopter and fixed-wing air medical | D3 context | 1,228 island missions over 9 years | Characterizes long-run island aeromedical transport. [A] |
| 133 | Moeller 2021 | Denmark | Retrospective service analysis | HEMS island vs mainland missions | D3 context | 3-year national missions | Island missions differ in profile and logistics from mainland missions. [A] |
| 134 | You 2024 | China | Review and feasibility analysis | eVTOL for aeromedical rescue | D3 | Technology and scenario review | eVTOL prospects for prehospital, interfacility and emergency rescue use. [A] |
| 135 | Liu 2024 | China | Policy review | National air medical rescue system | D3 | National development analysis | Development status, gaps and recommendations for China's air medical rescue. [A] |
| 136 | Yu 2023 | China | Perspective | Shipborne unmanned helicopter | D3 | Maritime rescue scenarios | Application prospects for maritime emergency medical rescue. [A] |
| 137 | Zhu 2018 (Acad Emerg Med) | United States | Propensity-score cohort | HEMS to verified trauma centre | D3 context | Rural trauma cohort | Rural HEMS survival benefit, OR 2.69 (95% CI 1.21-5.97). [A] |
| 138 | Funder 2017 | Denmark | Before-after implementation cohort | Physician-staffed helicopter | D3 context | Trauma quality-of-life follow-up | Quality-of-life outcomes around PS-HEMS implementation. [A] |
| 139 | Risgaard 2020 | Denmark | Systematic review | PS-HEMS vs ground EMS | D3 context | 18 included studies | Overall favourable but mixed PS-HEMS outcome evidence. [A] |
| 140 | Vuorinen 2024 (BMC Emerg Med) | Finland | Prospective observational study | Ambulance-helicopter hybrid transfer | D3 | Thrombectomy candidates | Hybrid conveyance strategy operationally viable for thrombectomy transfer. [A] |
| 141 | Vuorinen 2024 (Scand J Trauma Resusc Emerg Med) | Finland | Resource-optimization analysis | Air transport of thrombectomy candidates | D3 | Remote and rural prehospital system | Air transport optimizes remote prehospital resource use. [A] |
| 142 | Ackermann 2024 | Finland | Scenario-based cost-effectiveness analysis | PS-HEMS vs ground EMS | D3 context | National scenario modeling | Maps conditions under which PS-HEMS is cost-effective. [A] |
| 143 | Vuorinen 2025 | Finland | Retrospective analysis | Helicopter plus ground combined transfer | D3 | Thrombectomy door-in-door-out times | Combined strategy did not prolong door-in-door-out times. [A] |
| 144 | Enomoto 2020 | Japan | Nationwide retrospective cohort | Physician-staffed helicopter vs ground | D3 context | Pediatric trauma cohort | Mortality association of PS-HEMS in pediatric trauma. [A] |
| 145 | Chou 2026 | Taiwan, China | Quasi-experimental education study | VR air-to-ground handoff training | D3 | Emergency nurses | VR handoff training improved learning for aeromedical reception; window boundary check. [A] |
| 146 | Michaels 2019 | United States | National-database review | Helicopter vs ground ambulance | D3 context | Transferred trauma patients | Survival outcomes in interfacility trauma transfer. [C] |
| 147 | Garner 2015 | Australia | Single-centre randomized controlled trial (HIRT) | Physician (helicopter) prehospital care | D3 context | Severe blunt head injury | Physician prehospital management vs paramedic-only care. [B] |
| 148 | Surman 2024 | United Kingdom | Systematic review | UAVs in prehospital emergency medicine | D4 | 44 included papers | Maps medical and technical UAV evidence for prehospital emergency medicine. [A] |
| 149 | Pulsiri 2021 | Thailand | Systematic review with bibliometrics | Drones in EMS | D4 | Scopus corpus | Bibliometric structure of EMS-drone research; indexing variants noted. [A] |
| 150 | Campbell 2024 | Australia | Systematic review with LDA topic modeling | Drone healthcare delivery | D4 | 290 included articles, 2010-2024 | Two macro-themes: time-critical vs non-time-critical medical delivery. [A] |
| 151 | Johnson 2021 | United States | Review | Drones in emergency medicine | D4 | Published literature | Future roles and system implications of emergency-medicine drones. [A] |
| 152 | Robakowska 2022 | Poland | Review | UAVs in prehospital security | D4 | Published literature | UAV possibilities for medical-emergency prehospital operations. [A] |
| 153 | Zhang 2025 | China | Cross-sectional acceptance survey | Medical-supply delivery drones | D4 | Medical professionals sample | Quantifies acceptance and its determinants among clinicians. [A] |
| 154 | Pulsiri 2025 | Thailand | Systematic review | Drones in emergency medicine | D4 | Published literature | Applications, legal challenges and future directions. [A] |
| 155 | Zheng 2025 | China | Review, Chinese literature | Drones in emergency medical rescue | D4 | Published literature | Progress including AED airdrop, temperature-controlled blood transport and disaster supply. [A] |
| 156 | Huang 2025 | China | Strategy analysis, scene-task-capability framework | Low-altitude aircraft in emergency rescue | D4 | Framework analysis | Gaps and deployment strategies for low-altitude emergency medical rescue aircraft. [A] |
| 157 | Lu 2023 | China | Review | UAVs in aviation emergency rescue | D4 | Published literature | Reviews UAV status and applications including medical rescue. [A] |
| 158 | Al-Wathinani 2023 | Saudi Arabia | Rapid literature review | Disaster and prehospital drones | D4 | Regional evidence base | Drone roles for disaster management and prehospital care in Saudi Arabia. [A] |
| 159 | Shapira 2022 | Israel | Combined acceptance-framework study | Public-health emergency drones | D4 | Technology-acceptance modeling | Integrated framework for drone acceptance in health emergencies. [A] |
| 160 | Rosser 2018 | United States | Comprehensive review | Surgical and medical drones | D4 | Published literature | Cross-domain surgical and medical drone applications. [A] |
| 161 | Jain 2021 | Canada/Belgium | Comparative simulation study | UAV scene assessment | D4 | Paramedic-student mass-gathering exercise | UAV scene assessment vs standard practice; volume and pages via PubMed. [A] |
| 162 | Karaca 2018 | Turkey | Experimental search-and-rescue study | UAS in mountain search and rescue | D4 | Simulated SAR scenarios | UAV located victims faster than ground teams; domain assignment for author review. [B-PDF] |
| 163 | Bhatt 2018 | United States | Review | Telemedicine drones | D4 | Published literature | Targeted telemedicine applications of UAVs. [C] |
| 164 | Konert 2019 | Poland | Legal and practical analysis | Emergency-medicine drones | D4 | EU and Polish regulatory frame | Practical and legal aspects of medical drone use. [C] |
| 165 | Truog 2020 | Multi-country LMIC settings | Community-perception study | Medical delivery drones | D4 | Program communities | Community perceptions can make or break delivery programs. [C] |
| 166 | Hiebert 2020 | Canada/United States | Scoping review | Healthcare drones in North America | D4 | Published literature | Maps North American health-drone applications and gaps. [C] |
| 167 | Jeyabalan 2020 | Multi-country | Multi-site qualitative study | Healthcare delivery drones | D4 | Program managers and field staff | Context-specific challenges, opportunities and ethics. [C] |
| 168 | Cawthorne 2020 | Denmark/Netherlands | Ethical-framework development | Public-healthcare drones | D4 | Value-sensitive design framework | Ethics framework spanning design through assessment. [C] |

*Abbreviations: AED, automated external defibrillator; ALS, advanced life support; CI, confidence interval; EMS, emergency medical services; GIS, geographic information system; HEMS, helicopter emergency medical services; ICER, incremental cost-effectiveness ratio; ISS, Injury Severity Score; OHCA, out-of-hospital cardiac arrest; QALY, quality-adjusted life year; RR, risk ratio; UAV, unmanned aerial vehicle; VTOL, vertical take-off and landing.*
